# A yellow fever–Zika chimeric virus vaccine candidate protects against Zika infection and congenital malformations in mice

**DOI:** 10.1038/s41541-018-0092-2

**Published:** 2018-12-13

**Authors:** Dieudonné B. Kum, Niraj Mishra, Robbert Boudewijns, Ivan Gladwyn-Ng, Christian Alfano, Ji Ma, Michael A. Schmid, Rafael E. Marques, Dominique Schols, Suzanne Kaptein, Laurent Nguyen, Johan Neyts, Kai Dallmeier

**Affiliations:** 1 KU Leuven Department of Microbiology and Immunology, Rega Institute, Laboratory of Virology and Chemotherapy, Leuven, Belgium; 2 GIGA-Neurosciences, Interdisciplinary Cluster for Applied Genoproteomics (GIGA-R), University of Liège, C.H.U. Sart Tilman, Liège, Belgium; 3 Brazilian Biosciences National Laboratory (LNBio), Brazilian Center for Research in Energy and Materials (CNPEM), Campinas, Sao Paulo, Brazil

## Abstract

The recent Zika virus (ZIKV) epidemic in the Americas led to an intense search for therapeutics and vaccines. Here we report the engineering of a chimeric virus vaccine candidate (YF-ZIKprM/E) by replacing the antigenic surface glycoproteins and the capsid anchor of YFV-17D with those of a prototypic Asian lineage ZIKV isolate. By intracellular passaging, a variant with adaptive mutations in the E protein was obtained. Unlike YFV-17D, YF-ZIKprM/E replicates poorly in mosquito cells. Also, YF-ZIKprM/E does not cause disease nor mortality in interferon α/β, and γ receptor KO AG129 mice nor following intracranial inoculation of BALB/c pups. A single dose as low as 1 × 10^2^ PFU results, as early as 7 days post vaccination, in seroconversion to neutralizing antibodies and confers full protection in AG129 mice against stringent challenge with a lethal inoculum (10^5^ LD_50_) of either homologous or heterologous ZIKV strains. Induction of multi-functional CD4^+^ and CD8^+^ T cell responses against ZIKV structural and YFV-17D non-structural proteins indicates that cellular immunity may also contribute to protection. Vaccine immunogenicity and protection was confirmed in other mouse strains, including after temporal blockade of interferon-receptors in wild-type mice to facilitate ZIKV replication. Vaccination of wild-type NMRI dams with YF-ZIKprM/E results in complete protection of foetuses against brain infections and malformations following a stringent intraplacental challenge with an epidemic ZIKV strain. The particular characteristic of YF-ZIKprM/E in terms of efficacy and its marked attenuation in mice warrants further exploration as a vaccine candidate.

## Zika virus: Zika virus chimeric vaccine

Zika virus (ZIKV) infection generally results in mild symptoms but can cause serious developmental abnormalities in infants born to ZIKV infected mothers. Kai Dallmeier and colleagues at the KU Leuven in Belgium, engineered a chimeric live-attenuated vaccine (YF-ZIKprM/E) by swapping the glycoprotein from the Yellow Fever vaccine YFV-17D with that of a pre-epidemic ZIKV strain. YF-ZIKprM/E is very well tolerated with no adverse effects even following high dose intracranial infection. Mice highly susceptible to ZIKV infection—including AG129 and type I interferon receptor deficient strains—vaccinated with a single dose of YF-ZIKprM/E are fully protected from lethal ZIKV challenge. Protection can be achieved within 7 days and by low doses of YF-ZIKprM/E, is durable and generally results in sterilizing immunity. YF-ZIKprM/E elicits both neutralizing antibodies and robust cellular immunity. Finally, YF-ZIKprM/E can also prevent vertical transmission of ZIKV and achieve efficient protection of pups from neurological defects following intraplacental challenge.

## Introduction

The recent outbreak of the ZIKV in the Americas^1^ affected over 80 countries. The World Health Organization (WHO) declared the epidemic a global health emergency in particular because of the high rates of ZIKV-induced congenital malformations. The ZIKV is a flavivirus; several other medically important pathogens such as the dengue (DENV), the Japanese encephalitis (JEV), the tick-borne encephalitis (TBEV), the West Nile (WNV), and the yellow fever viruses also belong to this genus. The live-attenuated yellow fever virus, YFV-17D vaccine, developed by Max Theiler in 1937^2^ is a paradigm of an efficient and safe flavivirus vaccine. Vaccination with YFV-17D results in a rapid induction of (life) long-lasting protective immunity against YFV following a single dose of the vaccine. Since its discovery, the vaccine has been administered to an estimated 600 million people worldwide^3^ and is, based on its legacy track record, generally considered safe with only rare (~1 per million) serious adverse side effects [i.e., in particular vaccine-associated viscerotropic disease] limited primarily to specific risk groups.^4^ Several YFV-17D-based chimeric vaccines against the JEV^5,6^ (Imojev®), the DENV^7^ (Dengvaxia®), and the WNV^8^ (ChimeriVax-WN02) have been constructed wherein the prM/E genes of YFV-17D are replaced with each of the corresponding genes of the stated viruses.

Flaviviruses have a positive-sense single-strand RNA genome of ~11,000 nucleotides, which contains a 5′-untranslated region (UTR), a long open reading frame (ORF) and a 3′ UTR. The ORF encodes a single polyprotein precursor that comprises three structural (capsid [C], precursor membrane [prM], and envelope [E]) and seven non-structural (NS1–5) proteins. The structural proteins form, together with the viral RNA, the infectious viral particles whereas the NS proteins mediate viral replication but are for example also involved in the evasion and modulation of immune responses in infected cells.^9^ The signal peptide at the C terminus of the C protein (C anchor domain, Canch) regulates flavivirus packaging through coordination of sequential cleavages at the N terminus (by the viral NS2B/NS3 protease) and C terminus (by host signalases in the endoplasmic reticulum [ER] lumen) of the signal peptide sequence.^10,11^ Mutational studies demonstrate the importance of the signal sequence in viral particle formation and thus replication.^12,13^ Likewise, the assembly of viral particles can be markedly impaired when the prM/E genes of a particular flavivirus are exchanged in frame with the C-signal peptide of another flavivirus (Supplementary Fig 1). Whether a chimeric construct should contain the signal sequence of either the parental or the inserted virus is unpredictable, since efficient processing of the polyprotein and viral fitness do not necessarily correlate.^12,14,15^

**Fig. 1:**
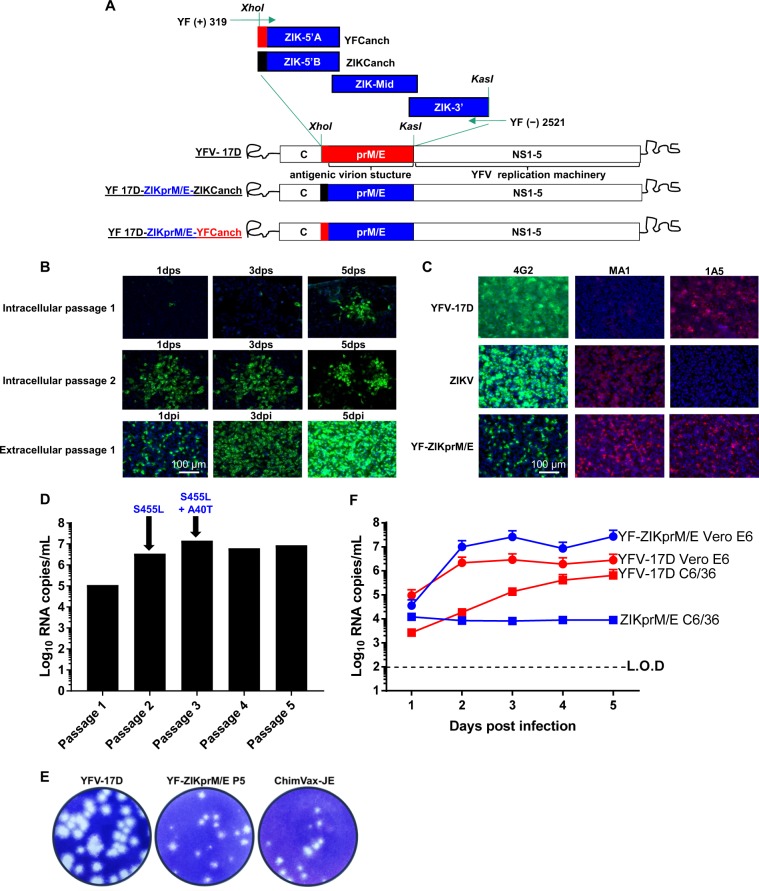
Construction and propagation of YF-ZIKprM/E. **a** Schematic representation of the YF-ZIKprM/E construction**:** ZIK-5′A contains the capsid anchor (C anch) sequence of YFV-17D whereas ZIK-5′B contains the C-signal sequence of ZIKV. ZIKV Yap (2007) sequence nt 313 to 2382 was cloned into YFV-17D between nucleotides 422 and 2451. **b** Immunofluorescence assay at selected time points after transfection of Vero E6 cells with YF-ZIKprM/E plasmid. At 2, 8, and 14 days post transfection (dpt), cultures were split and transferred to larger flasks and further cultured (Supplementary Table 1). After each split, a part of the cells was seeded in 8-well chamber slides and stained for viral antigen using pan-flavivirus monoclonal antibody (mAb) 4G2 (at 1, 3, and 5, days post split [dps]). Upper panels represent intracellular passages 1 and 2, respectively. Supernatant of passage 4 was used to infect naïve Vero E6 cells and virus infection and spread was monitored at indicated time points (bottom panel). **c** Antigenicity of YF-ZIKprM/E: Vero E6 cells were infected with either YFV-17D, ZIKV BeH819015,^45^ or YF-ZIKprM/E at multiplicity of infection (MOI) of 0.1 and stained 2dpi with pan-flavivirus antibody 4G2, anti-YFV NS1 mAb 1A5 or JEV E-specific mAb MA1-71256 that shows strong cross-reactivity only with ZIKV and YF-ZIKprM/E, but not with the YFV E protein. **d** Extracellular passaging of YF-ZIKprM/E in Vero E6 cells and quantification of viral RNA. Arrows indicate the first detection of ZIKV E protein mutations S455L and A40T, respectively. **e** YF-ZIKprM/E chimeric virus (Passage 5) forms smaller plaques (developed and stained 7dpi; complete well of a 6-well plate shown: diameter = 34.8 mm) on BHK-21J cells than the parental YFV-17D, similar to those formed by ChimeriVax-JE. **f** Replication kinetics of YFV-17D (red circles) and YF-ZIKprM/E (blue squares) on mammalian (Vero E6) and mosquito (C6/36) cell lines. Cells were infected with YFV-17D and YF-ZIKprM/E (Passage 5) at MOI of 0.01 and extracellular progeny viral RNA was quantified over time by qRT-PCR. Data are presented as mean values with error bars indicating SEM of *n* = 2 replicates. Dotted line denotes the limit of detection (L.O.D) of the qRT-PCR assay

While chimeric constructs based on the backbone of a non-attenuated DENV-2 strain D2Y98P or virulent ZIKV Cambodian strain FSS13025 have been generated,^16^ it is generally believed that the determinants of attenuation are largely associated with the backbone of the construct.^17^ Considering both, the high safety profile of YFV-17D^17^ and the neurovirulent properties of ZIKV^18^ from recent epidemics, we engineered (employing our proprietary DNA-YFV-17D plasmid, WO2014174078A1, Supplementary Fig 2a) a chimeric YF-ZIKprM/E construct (Fig. 1a) by swapping the sequence that encodes for the antigenic surface glycoproteins of YFV-17D with that of a prototypic Asian lineage ZIKV strain (isolated from the Yap Island in 2007). This pre-epidemic ZIKV strain does not carry the serine-to-asparagine (S139N) mutation in prM that has been found dominating in ZIKV outbreaks after 2013.^19^ Strains with this S139N mutation have been associated with increased ZIKV infectivity for both human and mouse neuronal progenitor cells and have been linked to neurovirulence (microcephaly) and mortality in foetuses.^18^ The chimeric virus consists of the antigenic determinants (prM and E) plus the capsid anchor sequence of ZIKV and the replication machinery (NS1–5), and UTRs of the YFV-17D^6^ (Fig. 1a and Supplementary Fig. 3).

## Results

### Construction and rescue of chimeric YF-ZIKprM/E

Following transfection of Vero E6 with the YF-ZIKprM/E construct (Fig. 1b) or a mCherry-tagged reporter virus variant thereof (Supplementary Fig 2a), replication of the chimeric virus appeared to be restricted to (some of) the transfected cells (Fig. 1b and Supplementary Fig 2b, c). However, no spread to neighboring cells was noted nor was virus released in the culture supernatant [as demonstrated by lack of detectable virus RNA by RT-PCR (limit of detection ~100 viral RNA copies/ml)] of cell culture supernatants harvested daily after transfection. This chimeric virus appeared to be replication-deficient with an apparent block prior to virus release. We hence explored strategies to propagate this virus (Supplementary Fig. 3). Firstly, classical approaches of either (a) serial passaging of culture supernatants of transfected cells in tissue culture or (b) intracranial inoculation and passaging in the brain of mice failed to yield measurable virus progeny neither by virus isolation, nor by causing any measurable signs of disease. Here we also explored various modifications to the chimeric YF-ZIKprM/E construct (variants) so that the virus carried the capsid anchor (C-signal sequence) of either YFV-17D or that of ZIKV (Supplementary Fig. 1). However, supernatant of cells transfected with either constructs did not cause any morbidity such as behavioral changes or weight loss in highly susceptible AG129 mice,^20,21^ neither following intracranial inoculation of 6-weeks-old mice (Supplementary Fig. 2d). In a further attempt to overcome the abortive infection, we explored whether subculturing of cells transfected with the cDNA construct would ultimately result in spread of the chimeric virus. Remarkably, following already one passage of subculturing of the transfected cells, foci of infected cells were observed 7 days post transfection (Fig. 1b). Continued subculturing for in total four passages (Supplementary Table 1) of the infected/transfected cultures resulted in a further spread of replicating virus (Fig. 1b, intracellular passage 1–2 and extracellular passage 1). The chimeric nature of the construct was confirmed by Sanger sequencing and by differential staining for the different antigens using YFV and ZIKV-specific monoclonal antibodies (Fig. 1c). Emergence of detectable amounts of chimeric virus in the culture supernatant, finally allowed further propagation of the virus to relatively high titers (approximately 1 × 10^7^ genomic copies/ml) in Vero E6 cells (Fig. 1d). The resulting virus had acquired two mutations in the E gene leading to a serine-to-leucine (S455L) and an alanine-to-threonine (A40T) change in the E protein [amino acid positions according to Theys et al.^22^]. (Supplementary Fig. 5 and Supplementary Table 2). Reverse engineering of these tissue culture adapted (TCA) mutations into the non-adapted virus (NAV) (Supplementary Fig. 6a) or the mCherry-tagged variant thereof resulted in the rescue of the otherwise replication incompetent chimeric virus to full infectivity (Supplementary Fig. 6b).

### Attenuation of chimeric YF-ZIKprM/E

Insufficient attenuation poses a safety risk for live vaccines. Therefore YF-ZIKprM/E was compared to the licensed YFV-17D. A reduced plaque size phenotype of a virus typically serves as a proxy for attenuation. The plaques formed by YF-ZIKprM/E virus on BHK-21J cells were smaller than those formed by YFV-17D and were comparable in size to those formed by the safe chimeric ChimeriVax-JE (Fig. 1e). YFV-17D replicates poorly in C6/36 mosquito cells (i.e. less efficiently than in mammalian cells) in contrast to wild-type YFV.^23^ Intriguingly, YF-ZIKprM/E, in contrast to YFV-17D, did not result in measurable amplification in C6/36 cells (Fig. 1f). This suggests that vector-borne transmission of this new live-attenuated vaccine candidate can be considered unlikely (as is also the case for YFV-17D). Next, when AG129 mice^20^ were inoculated intraperitoneally (i.p.) with a high dose of 1 × 10^6^ PFU of YF-ZIKprM/E, no observable signs of disease such as behavioral changes (hunched posture, ruffled fur, agitation/lethargy) or CNS involvements (such as paralysis) was noted during a 28 day follow-up period. However, when mice were injected with a much lower inoculum (1 × 10^3^ PFU or 10^4^-fold lower) of the parental YFV-17D, a rapid and consistent induction of disease necessitating euthanasia was observed (Supplementary Fig. 2E). Intracranial inoculation of 5-days-old BALB/c mouse pups with 1 × 10^4^ PFU of YF-ZIKprM/E did not result in any signs of diseases or mortality. This is in stark contrast to YFV-17D since intracranial inoculation of pups with as little as 10 PFU of this virus results in marked growth retardation (Supplementary Fig. 8a, b) and severe disease requiring euthanasia (mean day to euthanasia, MDE 9 ± 1days). YF-ZIKprM/E is thus at least 10^6^-fold attenuated over YFV-17D in adult AG129 mice and at least 10^3^-fold following intracranial inoculation in BALB/c pups. Although BALB/c pups intracranially inoculated with YF-ZIKprM/E did not show any signs of disease, replication of the virus in the brain was confirmed by means of qRT-PCR (Supplementary Fig. 8c). Interestingly, no emergence of variants that carry the neurovirulent prM S139N mutation^18^ was noted in the brains of these mice (Supplementary Fig. 8d).

### Immunogenicity and efficacy of YF-ZIKprM/E against lethal challenge in a stringent AG129 mouse model

**Fig. 2:**
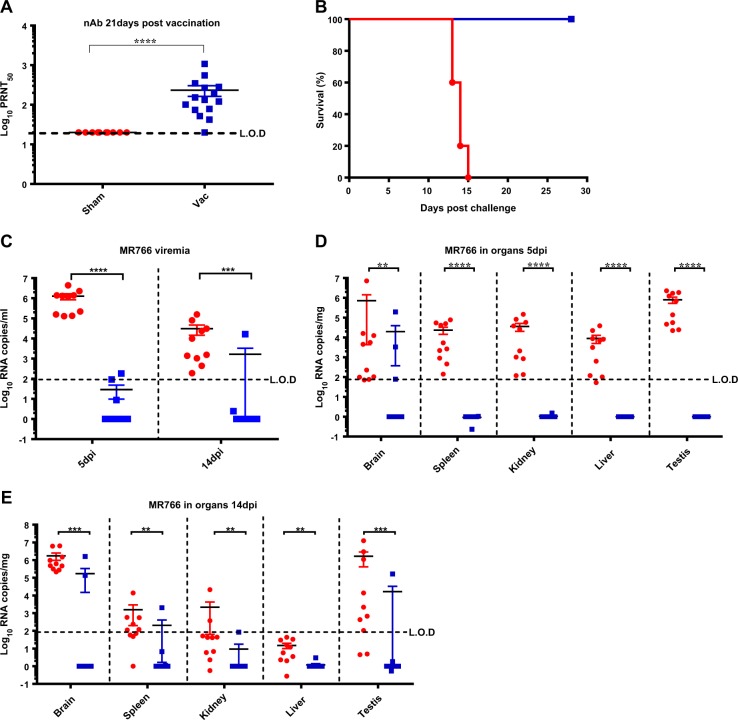
In vivo immunogenicity of YF-ZIKprM/E and protection of AG129 mice against lethal challenge with ZIKV MR766. **a** Seroconversion of vaccinated mice: AG129 mice (*n* = 15) were inoculated with 1 × 10^4^ PFU of YF-ZIKprM/E and the titers of ZIKV-specific neutralizing antibodies were assessed in serum at 21 days post immunization by PRNT (blue squares). Data compiled from three independent experiments with *n* = 5 per group. Sera from sham-vaccinated (*n* = 10) as controls (red circles). **b** ZIKV MR766 challenge: Twenty-one days post vaccination, vaccinated (blue squares, *n* = 5) and sham-vaccinated (red circles, *n* = 5) mice were challenged with 1 × 10^4^ PFU of ZIKV MR766. Vaccinated mice were monitored for 28 days, and remained healthy until the end of the experiment. Protection from ZIKV viremia **c** and virus dissemination to target organs **d**, **e** after challenge of vaccinated (blue squares, n = 10) and sham-vaccinated (red circles, *n* = 10) AG129 mice. Blood and organs were collected at day 5 **d** and 14 **e** post challenge. The subset of mice represented in **c**–**e** were not included in the survival analysis **b** because animals needed to be sacrificed earlier for organ collection. However, at euthanasia day 14 post challenge all sham-vaccinated mice showed signs of severe ZIKV-induced disease, as well as that particular YF-ZIKprM/E vaccinated animal that failed to seroconvert prior to challenge **a** resulting in viral break-through (outliers in **c** and **e**). Data are presented as mean values with error bars indicating SEM from two independent experiments (*n* = 5 each). Mann–Whitney two-tailed test was used for all statistical calculations. *P* values < 0.05 were considered statistically significant. *P < 0.05, **P < 0.01, ***P < 0.001. Horizontal dotted lines denote the limit of detection (L.O.D) of the assay

To demonstrate the immunogenicity and efficacy of YF-ZIKprM/E, adult AG129 mice were used; these are known to be highly susceptible following peripheral inoculation with minute amounts of ZIKV (10 PFU and less^21^). Mice were vaccinated i.p. with 1 × 10^4^ PFU of YF-ZIKprM/E or sham (MEM medium containing 2% FBS, the same medium used to prepare virus stock). In line with anticipated vaccine safety, all vaccinated mice remained healthy without weight loss. On day 21, blood was collected after which mice were challenged via the i.p. route with 1 × 10^4^ PFU (corresponding to ~10^4^ LD_50_) of the ZIKV strain MR766.^21^ In total *n* = 15 mice (three independent experiments) were vaccinated of which all but one (95%) seroconverted to virus neutralizing antibodies (nAb) with PRNT_50_ titers between 1:60 and 1:1235 [log_10_ geometric mean titer (GMT) 2.4] (Fig. 2a). Animals were monitored daily for weight loss and visible signs of disease. Five successfully immunized animals were challenged with 1 × 10^4^ PFU of ZIKV MR766; these mice were fully protected from virus-induced weight loss and mortality (Fig. 2b, e), whereas all sham-vaccinated mice (*n* = 5) developed ZIKV-induced disease and had to be euthanized (MDE 14 ± 1). The efficacy of the vaccine construct in protecting against challenge viremia and virus dissemination was assessed in a subset of vaccinated mice. Therefore vaccinated and sham-vaccinated mice (*n* = 10 each; two independent experiments) were euthanized 5 and 14 days post ZIKV MR766 challenge, respectively, and viral RNA load in different organs was quantified by means of qRT-PCR. Both at day 5 (peak viremia in sham-vaccinated mice) and 14 post challenge (at MDE in sham-vaccinated mice), viral RNA was undetectable in at least 8 out of 10 vaccinated mice (Fig. 2c). At day 14, the single mouse from the vaccine group without detectable anti-ZIKV antibodies prior to challenge [and that may have likely not been properly injected/vaccinated] had high viral RNA loads (Fig. 2c). Overall, there was an almost 4log_10_ reduction in serum viral load in vaccinated mice (Fig. 2c). Vaccination resulted also in complete reduction of viral loads in different organs including brain and testes at day 5 (Fig. 2d) and 14 (Fig. 2e) post challenge.

**Fig. 3:**
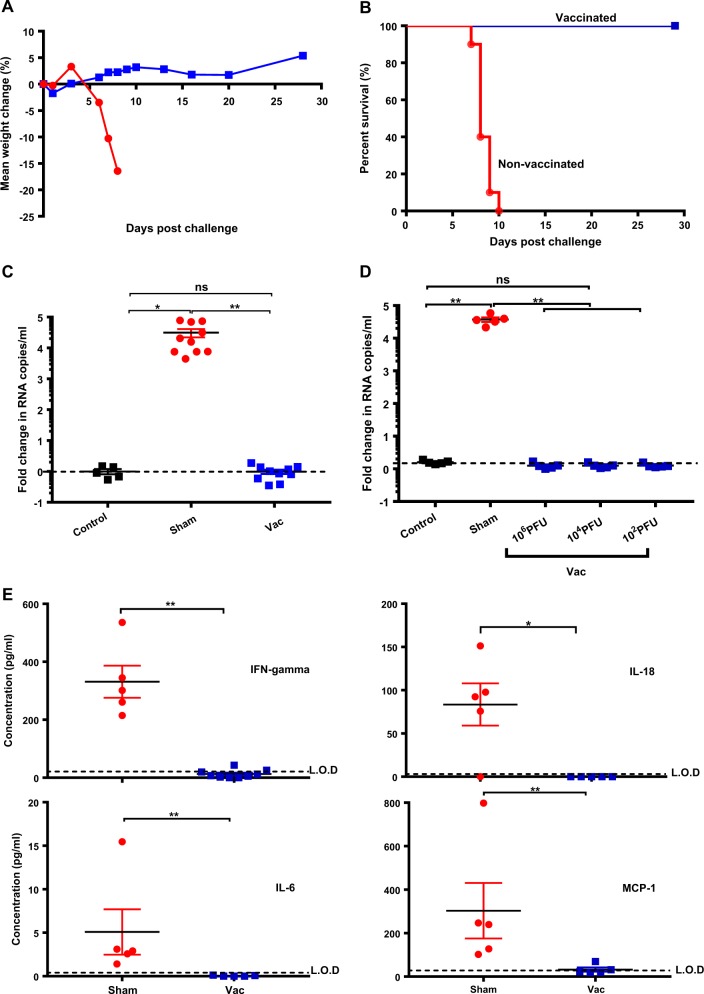
Protection of vaccinated AG129 mice against lethal challenge with ZIKV strain SL1602. AG129 mice were vaccinated (blue squares, *n* = 10) with 1 × 10^4^ PFU of YF-ZIKprM/E or sham-vaccinated (red circles, *n* = 10). Weight **a** and time to euthanasia **b** were monitored after challenge with either 1 × 10^3^ PFU or 1 × 10^5^ PFU of ZIKV SL1602. All vaccinated mice were grouped irrespective of the challenge dose. See Supplementary Fig. 9c and d for details about individual groups. **c** Protection from viremia in vaccinated AG129 mice: AG129 mice that had either been vaccinated with 1 × 10^4^ PFU of YF-ZIKprM/E (blue squares, *n* = 10) or that were sham-vaccinated (red circles, n = 10) were challenged with 1 × 10^5^ PFU or 1 × 10^3^ PFU of ZIKV SL1602 and viral RNA copy numbers were assessed at 3 days post challenge. All vaccinated mice were grouped irrespective of the challenge dose. See Supplementary Fig. 9c and d for details about individual groups. Sera from vaccinated mice collected pre-challenge served as controls. **d** AG129 mice were vaccinated with either 1 × 10^2^ PFU, 1 × 10^4^ PFU or 1 × 10^6^ PFU of YF-ZIKprM/E (blue squares, *n* = 5 per group) and were challenged with 1 × 10^5^ PFU of ZIKV SL1602 and ZIKV RNA copies in serum on day 3 post challenge were measured. Because of increased background values of qRT-PCR due to change in virus strain (MR766 to SL1602), values are reported as fold changed in RNA copies compared to serum RNA values of vaccinated mice prior to challenge. **e** Expression of cytokines 3 days post challenge: Vaccinated (closed squares, *n* = 5) and sham-vaccinated (closed circles, *n* = 5) mice were challenged with 1 × 10^5^ PFU of ZIKV SL1602 and levels of IFN-γ, IL-18, IL-6, MCP-1, and other cytokines (Supplementary Table 3) were assessed 3 days post challenge. Data are presented as mean values with error bars indicating SEM. Mann–Whitney two-tailed test was used for statistical analyses. Asterisk indicates a significant difference *P-values < 0.05, **P-values < 0.01. Background (dotted line) represents average RNA copies of vaccinated mice before challenge or the limit of detection (L.O.D) of the assay

Vaccine efficacy against lethal infection was also demonstrated when mice were challenged with the more aggressive South American (Suriname) ZIKV SL1602 isolate^24^ (Fig. 3a, b) following i.p. infection with either low (1 × 10^3^ PFU) or high (1 × 10^5^ PFU) titers of the virus (Supplementary Fig. 9a, b). This was corroborated by undetectable viral RNA (Fig. 3c) and infectious virus (Supplementary Fig. 9c) at peak viremia 3 days post challenge, even in mice vaccinated with a vaccine dose as low as 1 × 10^2^ PFU (Fig. 3d; for similar results using ZIKV MR766 see Supplementary Fig. 10a, b). Vaccinated mice did not show any challenge virus-induced elevation of serum cytokines (Fig. 3E) as demonstrated for IFN-γ, IL-18, IL-6, MCP-1, and others (Supplementary Table 3) that have previously been shown to be a sensitive marker of ZIKV replication and disease progression in the AG129 mouse model.^21^ Vaccination with YF-ZIKprM/E resulted already after 7 days in high titers of ZIKV-specific antibodies, the plateau phase was reached by day 10 (Supplementary Fig. 10c). All vaccinated mice (except 1 from the 21 day vaccination group which failed to seroconvert) were protected from an otherwise lethal challenge with 1 × 10^4^ PFU of a heterologous ZIKV MR766 strain (Supplementary Fig. 10d).

**Fig. 4:**
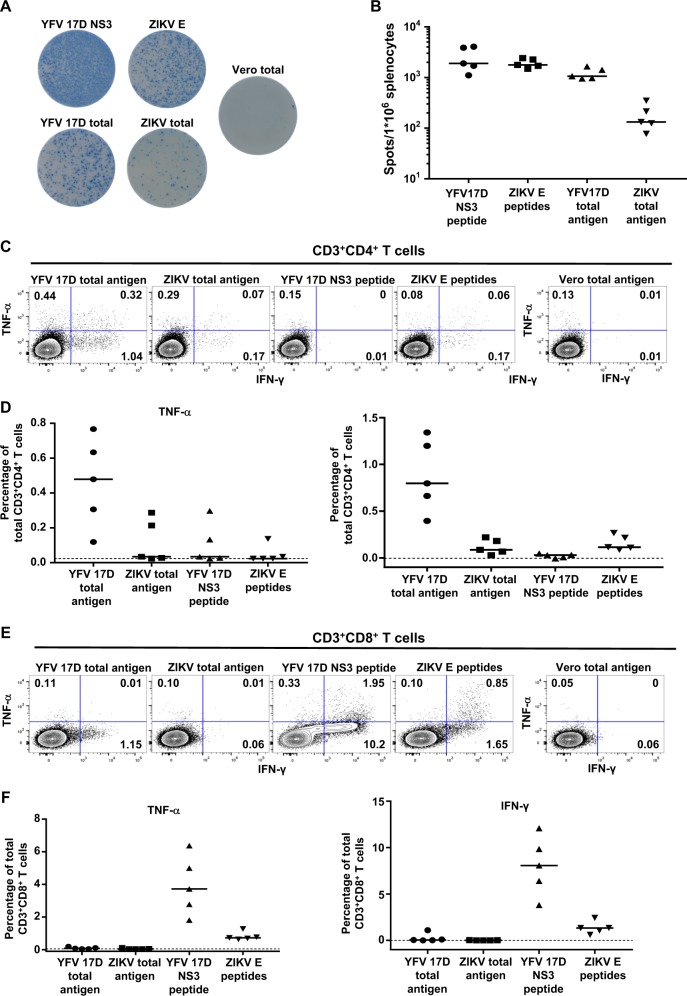
Analysis of cellular immunity (CD4^+^ and CD8^+^ T cells and cytokine response profiles) in vaccinated and sham-vaccinated AG129 mice. AG129 mice were vaccinated (*n* = 5, from two independent experiments) i.p. with 1 × 10^4^ PFU of YF-ZIKVprM/E. Ten weeks post vaccination, memory T cells were probed for either YFV-17D or ZIKV-specific recall responses in an ELISPOT. **a** Representative IFN-γ ELISPOT wells after overnight stimulation of 4 × 10^5^ splenocytes with the indicated antigen (complete well of a 96-well plate shown: diameter = 6.4 mm). **b** Spots per million splenocytes in IFN-γ ELISPOT after overnight stimulation with the indicated antigen. For each mouse, values are normalized by subtracting the number of spots in control wells (stimulated with Vero E6 cell lysate only). Median values are indicated with bars. **c** Intracellular cytokine expression levels of memory T cells in the spleen were analyzed 10 weeks after vaccination of 6–8-weeks-old male AG129 mice (*n* = 5, from two independent experiments) with 1 × 10^4^ PFU of YF-ZIKVprM/E. CD4^+^ T cells are defined as Zombie Aqua^−^ (ZA^−^), CD3^+^, and CD8^−^. **d** Representative TNF-α and IFN-γ expression profiles of CD4^+^ T cells following overnight stimulation with the indicated antigen. Numbers represent percentages of positive cells of the total CD4^+^ T cell population for each quadrant. **e** Intracellular cytokine expression levels of memory T cells. CD8^+^ T cells are defined as ZA−, CD3^+^, and CD8^+^. **f** Percentages of TNF-α-positive and IFN-γ-positive CD8^+^ T cells, normalized by subtracting the percentage of positive cells in corresponding control samples (stimulated with Vero E6 cell lysate). Data are presented as mean values with error bars indicating SEM. Background (dotted line) represents average values for sham-vaccinated mice

To assess the specificity of the cellular immune responses and to seek some evidence for longevity of the immunity, the magnitude of (memory) T cell responses elicited by YF-ZIKprM/E was studied several months after vaccination. ZIKV structural proteins are major targets of both CD4^+^ and CD8^+^ T cell responses.^25^ In the case of YFV-17D, structural as well as non-structural proteins have been shown to be involved in vigorous, broad and polyfunctional T cell responses both in mice and humans.^26^ AG129 mice were vaccinated with 1 × 10^4^ PFU of YF-ZIKprM/E and splenocytes were harvested 10 weeks after vaccination. YFV-17D and ZIKV-specific memory T cell responses were assessed by ELISPOT (Fig. 4a, b) as well as by intracellular cytokine staining (Fig. 4c–f). To that end splenocytes were incubated overnight with peptides derived from ZIKV E or with total ZIKV antigen (ZIKV-infected cell lysate) as recall antigens. This stimulation resulted in clear ZIKV-specific cellular responses, as is evident for the IFN-γ ELISPOT by a median (*n* = 5) increase (over background) of 1770 and 133 spots/10^6^ cells, respectively (Fig. 4a, b). Antigen-specific cytokine production was further assessed by the analysis of intracellular IFN-γ and TNF-α levels by means of flow cytometry (Fig. 4c–f). Results indicate that multi-functional CD8^+^ and CD4^+^ T cell responses with a cytotoxic Th1 polarization (Supplementary Fig. 11) may, in the case of YF-ZIKprM/E, contribute to protection against ZIKV challenge after vaccination. Of note, cellular responses were skewed towards the YFV-17D backbone (Fig. 4d, f and Supplementary Fig 11) but still showed a marked recall for ZIKV antigens, which is in contrast to the YFV-17D-based Dengvaxia® vaccine that has been reported not to result in pronounced DENV specific T cell immunity.^27^ Nevertheless, such results obtained in animal models, in particular in immunocompromised AG129 mice, should be interpreted with caution and may not per se allow to predict human cellular immune responses.

### Comparative potency of YF-ZIKprM/E in other mouse models

**Fig. 5:**
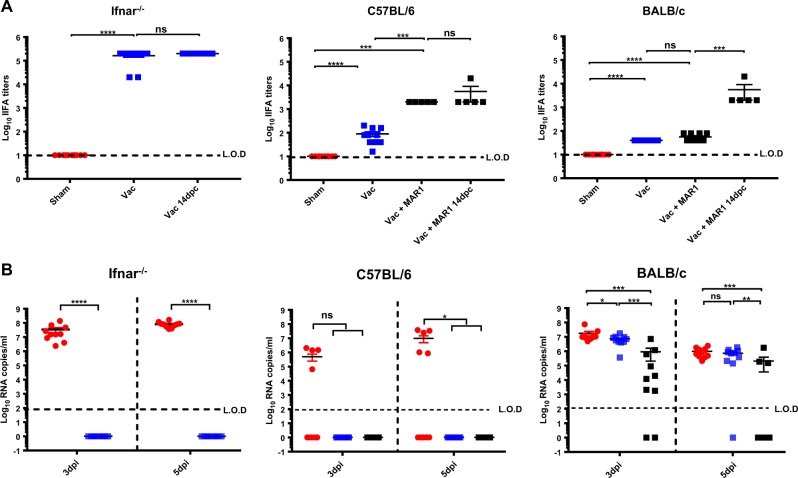
Antibody titers and protection from viremia in *ifnar*^−/−^, and wild-type mice upon ZIKV challenge. **a** Total antibody titers 28 days post vaccination. *Ifnar*^−^^/−^ mice were either sham-vaccinated with 2% FBS MEM (red circles, *n* = 10) or vaccinated with 1 × 10^4^ PFU of YF-ZIKprM/E (blue squares, *n* = 10) in groups of 5. C57BL/6 or BALB/c mice were either sham-vaccinated (red circles, *n* = 10) or vaccinated in the presence (black squares, *n* = 5 or 10) or absence (blue squares, *n* = 10) of an interferon blocking antibody, MAR1-A53. Mice were bled by submandibular puncture 28 days post vaccination and sera were used to quantify Ab titers by end point IIFA or viremia **b** at days 3 and 5 post infection. Data are presented as mean values with error bars indicating SEM. Mann–Whitney two-tailed test was used for statistical analyses. Asterisks indicate a significant difference: *P-values < 0.05, **P-values < 0.01, ***P-values < 0.001, ****P-values < 0.0001. Dotted lines denote the limit of detection of the assay

The seek further evidence for the efficacy of the construct and to exclude that the observations made in AG129 mice may have been largely biased by particular characteristics of these mice, we next assessed the effect of YF-ZIKprM/E in (i) immunocompetent wild-type BALB/c and C57BL/6 mice as well as in (ii) mice that are solely deficient in the interferon α/β receptor (*ifnar*^−/−^) (Fig. 5a, b). In brief, 4–6-weeks-old mice were either vaccinated with 1 × 10^4^ PFU YF-ZIKprM/E or were sham-vaccinated. For the ease of quantification, an indirect immunofluorescence assay (IIFA) was used as surrogate for PRNT to quantify ZIKV-specific Abs [IIFA end point titers correlated strongly with PRNT_50_ values (Supplementary Fig. 10e, f)]. All YF-ZIKprM/E vaccinated mice seroconverted to ZIKV-specific Abs with titers that were highly dependent on the mouse strain used (Fig. 5a). Vaccination of *ifnar*^−/−^ mice result rapidly in the induction of very high titers of antibodies that cause sterilizing immunity (no increase in ZIKV-specific Abs after challenge), and full protection against challenge viremia (Fig. 5a, b). Vaccination of immunocompetent C57BL/6 mice resulted in much lower titers of ZIKV-specific antibodies, but resulted in full protection against viremia as well (Fig. 5a, b). By contrast, although vaccination of BALB/c mice resulted in antibody titers that were comparable to those observed in C57BL/6 mice, there was little if any effect on challenge viremia in these mice (Fig. 5a, b). Generally, vaccine potency correlates directly with the extent with which the live-attenuated viruses replicate after inoculation. Therefore, the interferon receptor (IFNAR) blocking antibody MAR1-A53 was injected in an attempt to render C57BL/6 and in particular BALB/c mice more susceptible to YF-ZIKprM/E replication (and thus increase its immunogenicity). Pre-treatment of mice with MAR1-A53 before vaccination resulted in a significant increase in Ab titers in C57BL/6 mice, but not in BALB/c mice (Fig. 5a, b). Under this condition, sterilizing immunity was obtained in C57BL/6 mice, which was evident from the fact that there was no further increase in Ab titers after challenge. In MAR1-A53 pre-treated BALB/c mice, ZIKV Ab titers still raised significantly following challenge infection. A slight reduction in challenge viremia was however noted, and in some of the mice, no viremia was anymore detectable.

### Protection by YF-ZIKprM/E from ZIKV-induced congenital malformations

**Fig. 6:**
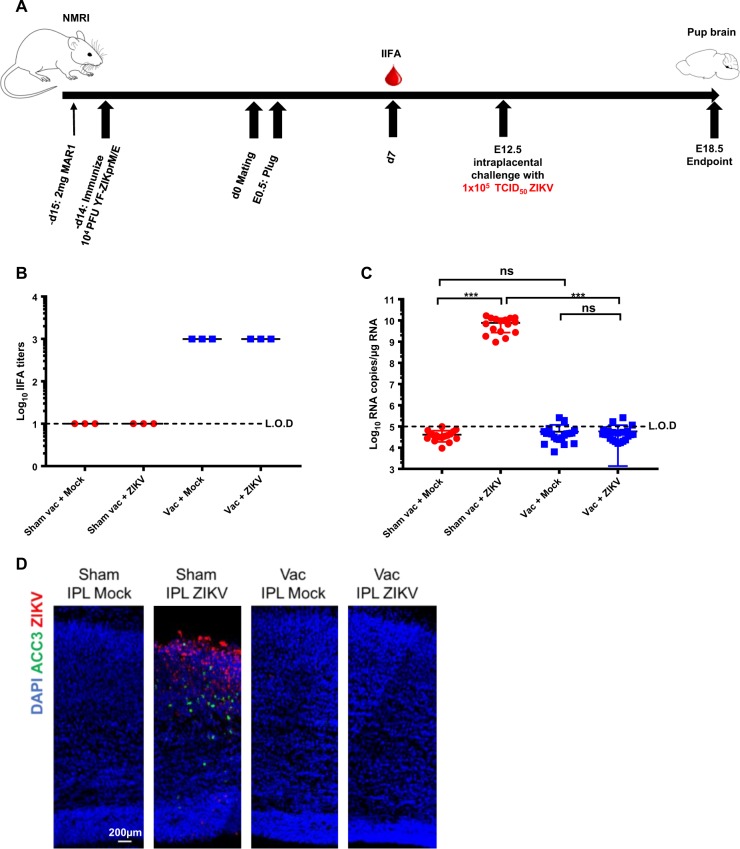
Protection from vertical transmission of ZIKV in NMRI mice in an intra-placental challenge model. **a** Schematic presentation of study outline. **b** Seroconversion (21 days post vaccination) of pregnant murine dams prior to intra-placental challenge with ZIKV. **c** Virus titers in fetal brains. qRT-PCR performed on total RNA extracts from mock- or ZIKV-infected E18.5 brains harvested from either sham-treated or vaccinated murine dams. The copy number of ZIKV was quantified and analyzed by two-sided Kruskal–Wallis test followed by Dunn post hoc comparison. Each group represents *n* = 16–22 biologically independent samples as indicated on respective scatter plots. Error bars show mean ± SEM. Levels of significance: ****P *<* 0.0001, ***P *<* 0.001, **P *<* 0.01, *P *<* 0.05 for all statistics herein; ns = not significant. **d** Confocal images of representative cortical sections from mouse embryonic E18.5 brains. Immunolabellings were performed for activated cleaved caspase-3 (ACC3) and ZIKV followed by counter-staining for nuclei in the green, red and blue channels, respectively. Sham = no vaccination of murine dams; Vac = vaccination of murine dams; IPL = Intra-placental injection at embryonic day 12.5; Mock = intra-placental challenge with mock media; ZIKV = intra-placental challenge with Zika virus (French Polynesia H/PF13, 1 × 10^5^ TCID50). Horizontal dotted lines denote the limit of detection (L.O.D) of the assay

It was next studied whether YF-ZIKprM/E protects against vertical transmission of the virus in a stringent intra-placental ZIKV challenge mouse model.^28^ Female wild-type NMRI mice were vaccinated, two weeks before mating, in the presence of MAR1-A53 Ab with either a single dose (1 × 10^4^ PFU of YF-ZIKprM/E) or with sham (Fig. 6a). Serum levels for ZIKV-specific Abs achieved in vaccinated dams at 21dpv (i.e. 5 days prior to challenge) (Fig. 6b) were comparable to the titers measured in C57BL/6 mice (Fig. 5a). Twelve days after conception (embryonic age E12.5), the developing embryos (16–22 per group) were challenged intra-placentally (IPL) with 1 × 10^5^ TCID_50_ of ZIKV strain (French Polynesia) H/PF13 as previously described.^28^ Six days later (E18.5) foetuses were analyzed. Pups from vaccinated mothers had undetectable levels (by qRT-PCR) of ZIKV RNA (>4log_10_ reduction; *n* = 16 sham-vaccinated/challenged; *n* = 22 vaccinated/challenged; *p* < 0.001) similar to non-challenged pups (*n* = 17 sham-vaccinated/mock-infected; *n* = 16 vaccinated/mock-infected). Activation of apoptotic caspase-3 (ACC3, Fig. 6d), and as consequence a general disorganization of the cortical layers and thinner upper layers, was only and consistently observed in ZIKV antigen positive brains of pups from sham-vaccinated dams following IPL challenge. Thus, vaccination with a single dose (1 × 10^4^ PFU) of YF-ZIKprM/E prior to pregnancy, conferred full protection against fetal infection and associated malformations, even when challenged by direct IPL inoculation thereby bypassing the feto-maternal barrier.

## Discussion

A number of ZIKV vaccine candidates have been developed employing various approaches and technologies,^29^ ranging from subunit DNA/RNA vaccines,^30,31^ virus-like particles^32,33^ and inactivated whole virus,^34^ over virus vectored^35^ to live-attenuated virus^36^, and chimeric virus^37^ vaccines. Several proved efficient in protecting against experimental challenge in mice and some also in non-human primates. Only a few candidates have been shown to be efficacious against vaccine challenge in very stringent interferon-α/β and γ-receptor-deficient (IFNAGR-KO) mouse models [AG129:^32–34^ AGB6:^38^], as well as in murine ZIKV pregnancy challenge models.^37,39^ To our knowledge, the vigorous intra-placental ZIKV challenge model^28^ has not been used before to assess protection from ZIKV-induced congenital malformations.

We generated a chimeric live-attenuated ZIKV vaccine candidate (YF-ZIKprM/E) by swapping the capsid anchor (Canch) and prM/E sequence of the YFV-17D vaccine virus with that of the corresponding sequence of a pre-epidemic Asian ZIKV strain. The ZIKV isolate that was used to this end carries a serine at position 17 in the ZIKV prM (position 139 in the ZIKV polyprotein)^22^, rather than the asparagine that has been reported to be associated with microcephaly. In contrast to other YFV-17D-based chimeric constructs,^5,40^ where the Canch sequence of the backbone virus was required for infectivity, we found that the Canch of ZIKV was needed here. In addition, the chimeric virus required adaptive mutations for efficient replication and extracellular release. Already during construction of the original chimeric cDNA construct, two presumably replication-enhancing mutations were adopted from the modified YFV-17D backbone of ChimeriVax-JE^6^ (Supplementary Fig. 4 and Supplementary Table 2). Another two mutations were consecutively acquired after intracellular passage of the virus. The two adaptive mutations (G1097A resulting in A40T and C2343T resulting in S455L) in the E gene^22^ were shown, by reverse engineering, to be sufficient and required for increased infectivity and propagation of the vaccine virus to higher titers (Supplementary Fig. 6). These mutations likely contribute to unlocking a block of the chimeric YF-ZIKprM/E at the level of virus release. S455L is located immediately upstream of the transmembrane anchor domains (TM) 1 and 2 of the E protein. TM1 and TM2 are involved in regulation of flaviviral particle maturation^41^ and function as endoplasmic reticulum retention signal.^42^ A threonine is commonly found at position 40 in current ZIKV strains; this T40 residue is located at the surface of the E protein, but is not involved in Ab mediated neutralization.^43^ Nevertheless, the two amino acid changes were found to also enhance the infectivity of YF-ZIKprM/E in a panel of human cell types that are known to support ZIKV replication (Supplementary Fig. 7). YF-ZIKprM/E has a smaller plaque phenotype than YFV-17D, and unlike the parental virus, replicates poorly in mosquito cells. Remarkably, and again in sharp contrast to YFV-17D, YF-ZIKprM/E does not cause disease when inoculated intracranially (even at very high titers) in BALB/c pups or systemically in adult (IFNAGR-KO) AG129 mice. The remarkable attenuation of YF-ZIKprM/E may be a beneficial characteristic when further development as a vaccine is envisaged. Poor replication in mosquito cells may be an additional asset [although there is no evidence that the parent YFV-17D (which replicates in mosquito cells) can be transmitted by mosquitos].

We first assessed the effect of the YF-ZIKprM/E in a stringent ZIKV infection model in AG129 mice. Even a minute inoculum of the ZIKV (and of most other flaviviruses), results rapidly in disease and mortality in these mice.^21^ Vaccination of AG129 mice with YF-ZIKprM/E results in rapid seroconversion (within 7 days) to high levels of virus neutralizing antibodies. These mice are protected against ZIKV viremia and virus-induced disease both following homologous and heterologous challenge. Overall, sterilizing immunity was obtained in most animals, with a ~4log_10_ reduction in serum viral load and no detectable viral RNA in various organs (including brain and testes). Even an inoculum as low as 10^2^ PFU of YF-ZIKprM/E proved sufficient to result in protective immunity. For YFV-17D, both in mice and in humans, structural as well as non-structural proteins have been shown to be involved in vigorous, broad and polyfunctional T cell responses^26^. We observed clear ZIKV-specific cellular responses that are multi-functional (CD8^+^ and CD4^+^ T cell responses with a cytotoxic Th1 polarization) in nature. Even though cellular responses were skewed towards the YFV-17D backbone, a marked recall for ZIKV antigens was noted. This is in contrast to the observations made for Dengvaxia® vaccine^27^.

To exclude that the observations made in the interferon receptor KO AG129 mice are the results of an aberrant mouse strain/immune system, we assessed the effect of YF-ZIKprM/E in immune competent mice (C57BL/6, BALB/c and NMRI) as well as in interferon α/β receptor (*ifnar*^−/^^−^) KO mice. Vaccination with YFV-ZIKprM/E resulted in consistent potency in wild-type C57BL/6 mice and in NMRI mice (for the latter see *infra*) Only in BALB/c mice, the chimeric vaccine largely failed to induce protective immunity. When the interferon receptors were blocked pharmacologically (using an antibody) in BALB/c mice, at least some of the mice (70%) had undetectable levels of viremia after challenge. When immunocompetent NMRI mice dams were vaccinated with YFV-ZIKprM/E (in the presence of the interferon receptor blocking antibody) their fetuses were completely protected against a stringent intra-placental challenge with the ZIKV (at day 12 after conception; embryonic age E12.5). No viral RNA was detectable nor were any malformations observed. In three other studies in which the effect of vaccination of murine dams [with, respectively, two live-attenuated^38,39^ and one chimeric ZIKV vaccine^37^] on their foetuses was studied, either a non-sterilizing reduction in viral loads in the embryo heads was observed^39^ or was not reported.^37,38^ All three vaccine candidates prevented however congenital malformations. It should be noted that the model of direct intra-placental inoculation as used in the present study, is likely more vigorous than the peripheral challenge models used before;^37,39^ yet YFV-ZIKprM/E was able to completely prevent infection in the foetuses.

In conclusion, we generated a chimeric live-attenuated ZIKV vaccine candidate (YFV-ZIKprM/E) by swapping the Canch and prM/E sequences of a pre-epidemic Asian ZIKV into the YFV-17D backbone (that carried itself two mutations from the Imojev® construct). The resulting virus was initially over-attenuated, but acquisition of two tissue culture adaptive (TCA) mutations allowed to amplify the chimeric virus to high titers. This virus is, in stark contrast to the parent virus YFV-17D, avirulent in AG129 mice as well as following intracranial inoculation in mouse pups. In contrast to YFV-17D, YFV-ZIKprM/E replicates poorly in mosquito cells. The virus is highly effective in inducing a protective immune response in various mouse models (including immunocompetent C57BL/6 and NMRI mice, but only partially effective in BALB/c mice) and fully protects foetuses of vaccinated NMRI dams against direct intra-placental ZIKV challenge. During revision of the current study, another chimeric YFV-17D-based ZIKV vaccine candidate (ChimeriVax-Zika, CYZ) was reported.^44^ The immunogenicity and efficacy of CYZ (partial protection against challenge viremia) has been demonstrated in A129 (IFNAR-KO) mice. The molecular basis of the remarkable differences in the potency of YF-ZIKprM/E versus CYZ warrants further investigation. Reasons for such differences may include (i) the actual choice of the prM/E sequence (Yap 2007 *vs*. French Polynesian 2013 ZIKV), (ii) the adaptive TCA mutations acquired in YF-ZIKprM/E (Fig. 1d and Supplementary Fig. 5 and 6), or (iii) the particular replication-enhancing mutations in the backbone of YF-ZIKprM/E [derived from ChimeriVax-JE^6^ [Supplementary Fig. 4], or (iv) combinations thereof. The particular characteristics of YF-ZIKprM/E (in terms of efficacy and safety in the models used here) warrants further exploration of this chimeric virus as a vaccine candidate.

## Methods

### Plasmid construction

Plasmid pShuttle/YFVax is an inducible BAC and derivative from the synthetic construct as disclosed in WO2014174078 that was constructed using standard recombinant DNA methods. Plasmid pShuttle/ChimeriVax-JE was designed to contain the prM/E sequence of the Japanese encephalitis vaccine SA14–14–2 plus two additional adaptive (missense) codon changes in the YFV-17D NS2A and NS4B regions^6^ containing an additional *Kas1* site at the end of the prM/E coding sequence (Fig. 1a). Furthermore, pShuttle/ChimeriVax-JE contains silent *Xho1* (mutations 3), *BstE2* (mutation 4), and *Nhe1* (mutation 5) restriction sites in the YFV-17D sequence as depicted in supplementary Fig 1A and not present in the original ChimeriVax-JE. The capsid anchor prM/E region of the ZIKV Yap isolate (EU545988) corresponding to the polyprotein open-reading (ORF) nt 313–2382 was synthetized by custom gene synthesis (Integrated DNA Technologies, Haasrode, Belgium) as three overlapping gene blocks namely (a) ZIK-5′A or ZIK-5′B flanked with YFV or ZIKV capsid anchor at their 5′ end, respectively. (b) ZIK-Mid and (c) ZIK-3′ flanked with NS1 sequences of YFV at their 3′ end. Blocks were assembled by fusion PCR using primers containing *Xho1* and *Kas1* restriction sites, which allowed ligation of the amplicons directly into the pShuttle/ChimeriVax-JE. The construction of the mCherry variants is described elsewhere (Schmid et al., manuscript in preparation). The resulting construct was ligated upstream of ZIKprM/E in the YF-ZIKprM/E construct described above.

### Cells

Vero E6 and BHK-21J cells used in this study were a generous gift from Peter Bredenbeek, LUMC, NL. Cells were maintained in Minimum Essential Medium (MEM-Rega-3, Invitrogen Life Technologies) supplemented with 10% FBS, 2 mM l-glutamine (Gibco, Belgium), 1× Anti-Anti (Streptomycin and Amphotericin B; Gibco Life Technologies, Belgium), 1% sodium bicarbonate (Gibco, Life Technologies, Belgium), and incubated at 37 °C, 5% CO_2_.

All other cell lines (see Supplementary Table 5) were maintained in the lab in their respective media.

Virus was generated and propagated in Vero E6 cells. To generate viruses from plasmid constructs, 5 × 10^5^ Vero E6 cells were transfected with 2.5 µg of plasmid DNA using TransIT®-LT1 transfection reagent (Mirus Bio LLC, Belgium) following manufacturer’s instruction. Two days post transfection (dpt), cells were washed twice with PBS, trypsinized and transferred to 25 cm^3^ flasks in a total volume of 5 ml of 2% FBS medium containing 1× Anti-Anti. This cycle was repeated three more times at day 8, 14, and 20 post transfection, each time expanding the cell substrate by providing increasingly larger tissue culture vessels (Supplementary Table 2). Cell culture was stopped after 25 days. Intracellular replication of chimeric viruses launched from the inducible BACs occasionally led to the production and release of fully infectious progeny virus particles into the tissue culture supernatant. To allow amplification of such chimeric virus as soon as it emerges, a fraction of the supernatant of the transfected cells was transferred to each next cell passage. Once induction of CPE was noted, culture supernatant was harvested, centrifuged at 3000 rpm at 4 °C, aliquoted and stored at −80 °C before further use. To prepare the virus stocks, YF-ZIKprM/E virus derived post intracellular passaging of dividing cells was passaged in Vero E6 cells and passages 3 (P3) and 5 (P5) were used for further studies.

### Viruses

ZIKV strains MR766,^21^ SL1602^24^, and BeH819015^45^ were obtained from the European Virus Archive (EVA) (EVA; http://www.european-virus-archive.com/viruses/zika-virus-strain-mr766), Prof. Martijn van Hemert, University of Leiden, The Netherlands and Prof. A. Merits, University of Tartu, Estonia.YFV-17D, Stamaril® (Sanofi-Pasteur) was passaged two times on BHK-21J cells before use.

### Animals

Mice deficient in both interferon-α/β and -γ receptors (AG129; B&K Universal, Marshall Bio resources, UK) were bred in-house at the Experimental Animal Facilities of the University of Leuven, Leuven, Belgium and randomly assigned for study. All experiments using mice strictly followed the Belgian guidelines for animal experimentation and the guidelines of the Federation of European Laboratory Animal Science Associations. The Ethical Committee of the Animal Research Center of the University of Leuven pre-approved all experiments (P140–2016). Throughout the study, 6–8 weeks old animals were vaccinated intraperitoneally (i.p.) with 1 × 10^2^–1 × 10^6^ PFU of YF-ZIKprM/E prior to i.p. challenge with 1 × 10^3^ PFU–1 × 10^5^ PFU of heterologous MR766^21^ or SL1602^24^ strain as essentially described.^21^ In addition, to determine the kinetics of protection post vaccination, mice were challenged at 0, 7, and 14 days post vaccination. Mice were monitored daily for signs of disease and mortality. Animals were euthanized when they showed overt signs of disease such as paralysis of hind limb, hunch posture, ruffled fur, watery/sunken eyes, and/or more than 20% weight loss. To determine viremia and virus dissemination to organs post challenge, vaccinated and non-vaccinated mice were euthanized on day 3, 5, and 14 post challenge and blood was collected and immediately incubated on ice after terminal heart or submandibular puncture, after which it was centrifuged at 3000 rpm for 15 min and stored at −80 °C. Similarly, various organs of these animals were harvested on dry ice with or without RNAlater (Thermo Fischer) post perfusion with PBS and stored at −80 °C.

BALB/c mice and pups were purchased from Janvier Labs, Le Genest-Saint-Isle, France. Pups (*n* = 5 per group) were intracranially inoculated with either 10 PFU of YFV-17D or 1 × 10^4^ PFU of YF-ZIKprM/E and monitored for morbidity and mortality for 21 days post inoculation.

*Ifnar*1^−/−^ mice were bred in-house at the KU Leuven animal facility, while BALB/c, C57BL/6 were purchased from Janvier Labs, Le Genest-Saint-Isle, France. Mice were vaccinated and treated as described above for AG129. Because immune competent mice do not readily replicate ZIKV,^46^ they were immunized in the presence or absence of 2 mg of an interferon blocking antibody, MAR1-5A3, administered i.p. 1 day prior to immunization. Twenty-one days post immunization, mice were bled by submandibular puncture and scored for the presence of antibodies in sera prior to i.p. challenge with 1 × 10^4^ PFU ZIKV MR766. Vaccinated and sham-vaccinated BALB/c and C57BL/6 mice were injected i.p. with 2 mg of MAR1-5A3 to render them susceptible to infection.^46^ Mice were bled on days 3 and 5 post challenge for RNA quantification by qRT-PCR.

Immunocompetent NMRI wild-type female mice (adult at 2–3 months of age) were purchased from Janvier Laboratories, France and treated as approved by the Animal Ethics Committee of the University of Liège (#16-1829). Timed-mated dams were housed in specific-pathogen-free facility under standard conditions, with ad libitum access to food and water. One day prior to i.p vaccination with either 1 × 10^4^ PFU YF-ZIKprM/E or 2% FBS MEM (mock), mice were administered 2 mg of MAR1-5A3. Fourteen (14) days post vaccination, mice were mated with NMRI males and mating plugs were observed the following day, embryonic day 0.5 (E0.5). At E12.5, pregnant females were operated upon (mini-laparotomy) and challenged intra-placentally (IPL) with 1 × 10^5^ TCID_50_ of ZIKV strain H/PF13. Incisions were sutured, and mice were observed for 6 days (E18.5) and euthanized thereafter to determine fetal infection and malformations. The intra-placental injections of embryos at E12.5 were performed as previously described^28^ with slight modifications. Surgeries were performed at noon, when the day after mating was considered E0.5. Pre-operative analgesia with Temgesic (buprenorphine 0.1 mg/kg body weight, Schering-Plough, Brussels, Belgium) was administered subcutaneously before induction of anesthesia with isoflurane (Abbot Laboratories Ltd, Kent, UK) in an oxygen carrier. Subsequently, a mini-laparotomy (1.0 to 1.5 cm incision) was performed through the lower ventral peritoneum. The uterine horns were carefully extracted and placed on warm humidified gauze pads.

### Virus growth kinetics

To assess the growth kinetics of YF-ZIKprM/E and YFV-17D on Vero E6 and mosquito cells (C6/36), cells were seeded over night at a density of 1 × 10^6^ cells per well of a 6-well plate in a total volume of 3 ml 10% FBS MEM or Leibovitz’s L-15 medium (supplemented with 10% FBS, 1% Penicillin/Streptomycin, tryptose phosphate broth, and nonessential amino acids), respectively. Vero E6 and C6/36 cells were washed twice with PBS prior to infection with 1 × 10^4^ PFU of either YFV-17D or YF-ZIKprM/E (MOI = 0.01) and incubated at 37 or 28 °C, respectively, for 1 h. Cells were again washed twice and 3 ml of either 2% FBS MEM or Leibovitz’s L-15 medium, respectively, was added and plates were incubated at appropriate temperatures. Overall, 250 µl of supernatants were harvested daily (and replaced with 250 µl fresh medium) for RNA extraction and quantification. All experiments were done in duplicate and data presented as mean ± SEM.

### RNA extraction and qRT-PCR

RNA extractions from serum and organs fixed in RNAlater were performed as described elsewhere.^21^

Quantification of Zika virus copy number in infected fetal mouse brains was done by qRT-PCR and calculated according to previously published protocol.^28^ Briefly, a standard curve was created by performing serial dilutions of the plasmid containing a 76-bp long sequence (inserted BamH1/Xho1) from the Zika genome (from nt 1086 to 1162) and querying these dilutions by qRT-PCR analysis. The following primers were used to create the standard curve: ZIKV 1,086 (genome pos. 1,086–1,102): 5′-CCGCTGCCCAACACAAG-3′; ZIKV: 1,162c (genome pos. 1,162–1,139): 5′-CCACTAACGTTCTTTTGCAGACAT-3′. The standard curve allows conversion of the Cp of samples into no. of Zika copies/μl. The following formula as previously described was used:

$${\mathrm{Number}}\,\left( {{\mathrm{No.}}} \right)\,{\mathrm{of}}\,{\mathrm{copies/\mu l}} \times k \times d \times P = {\mathrm{No.}}\,{\mathrm{of}} \,{\mathrm{copies/\mu g}}\,{\mathrm{RNA}}$$

where *k* is a correction factor introduced to calculate the no. of copies with respect to 1 μg of RNA (in this instance 0.5 μg of RNA was used to synthetize cDNA, so *k* *=* 2), *d* is a dilution factor, taking into account the dilution of cDNA used for the analysis (in this case the cDNA was diluted 1:30, so *d* *=* 30) and *P* is a correction factor introduced to calculate the no. of Zika copies with respect to the total amount of cDNA (here 3.4 μl in a total of 20 μl of cDNA mix were used, so *P* *=* 5.88). Finally, the no. of copies/μg RNA was divided for the relative concentration of each sample analyzed by qRT-PCR.

### Immunohistochemistry

Embryonic (E) E18.5 mouse brains were dissected in 0.1 M phosphate-buffered saline pH 7.4 (PBS) and fixed in 4% paraformaldehyde (PFA in PBS) for 1 h at room temperature (20 to 24 °C), followed by cryoprotection (30% sucrose in PBS) before embedding in gelatin for cryosectioning at 14 μm (Leica) onto slides (SuperFrost Plus, VWR International).

Fluorescent immunohistochemistry was performed as previously described.^28^ In summary, antigen retrieval (Dako Target Retrieval Solution) of mouse brains were performed at 95 °C for 15 min before incubation with primary antibodies. The following primary antibodies were used anti-cleaved-caspase-3 (1:100, rabbit, #9661, CST); anti-flavivirus group antigen (1:1000, mouse, MAB10216, Millipore). The respective secondary antibodies used were anti-mouse, and anti-rabbit antibodies, conjugated with Alexa Fluor-488 or Alexa Fluor-555 (Jackson ImmunoResearch Laboratories or Life Technologies) and diluted at 1:800.

Nuclei were counter-stained with DAPI (1:10,000, Sigma) and mounted in Mowiol (Sigma) solution.

### Plaque assay and plaque reduction neutralization test (PRNT)

Infectious virus titers in serum and organs were also determined by plaque assay. In brief, 1 × 10^6^ BHK-21J cells per well of a 6-well plate were seeded overnight in seeding medium, washed twice in PBS and incubated with serial dilutions of virus in assay medium for 1 h at 37 °C. Later, virus was removed, cells were again washed twice with PBS and overlaid with 0.5% low melting agarose (Invitrogen) prepared in overlay medium (MEM plus 2% FBS). Agar was allowed to solidify at room temperature prior to incubation at 37 °C for 7 days. Thereafter, cells were fixed with 4% formaldehyde and plaques were visualized by staining with 1% crystal violet for 5 min. Plaques were manually counted and reported as PFU/ml of virus stock. Virus neutralizing antibodies (nAb) are defined operationally by their ability to prevent virus infection in tissue culture and/or *in vivo* infection models. The presence of ZIKV-specific nAb was determined quantitatively by plaque reduction neutralization test (PRNT). For PRNT, 40–80 PFU of ZIKV MR766 were incubated for 1 h with a dilution series of sera prior to addition to BHK-21J cells as described above. For each immune serum a median PRNT (PRNT_50_) titer [determined in technical triplicates and expressed as geometric mean thereof] was defined as dilution at which the plaques count was reduced to less than 50% of the mean number of plaques from untreated virus inocula (obtained by back titration of inocula in the same experiment).

### Immunofluorescence assay (IFA)

Virus replication and spread was monitored using an in-house developed IFA. To achieve this, 300 µl of transfected cell suspension derived after trypsinization and resuspension in appropriate volume of MEM plus 2%FBS was transferred to wells in an 8-well Lab-Tek chamber slide (ThermoFisher Scientific, USA) and stained at indicated time points. Briefly, cells were washed twice with PBS, fixed with 4% paraformaldehyde for 20 min followed by two washes and permeabilization with 0.1% Triton X-100 for 5 min. Cells were again washed twice with PBS and covered with blocking buffer (1% FBS in PBS and 0.05% Tween-20). After 1 h of incubation at room temperature, blocking buffer was removed and 200 µl of pan-flavivirus mouse monoclonal antibody 4G2 (1:1000, mouse, MAB10216, Millipore) diluted in blocking buffer was added and the slide was rocked on a shaker at 300 rpm for 1 h. Cells were washed 3 times (5 min per wash on a shaker) with wash buffer (PBS + 0.05% Tween-20) and counter-stained with goat anti-mouse secondary antibody conjugated with a fluorescent probe, Alexa 488 (1:1000, goat anti-mouse, A11001, Life Technologies, USA). Secondary antibody was removed and the cells were again washed 3 times (5 min per wash on shaker) with wash buffer. Finally, cells were covered with a mounting medium that contained a DAPI stain for nuclear staining (blue) and observed under a fluorescent microscope (Floid Cell Imaging Station, ThermoFisher, Germany).

To detect ZIKV-specific antibodies in mouse serum, indirect IFA (IIFA) was used as described above with some modifications. Briefly, Vero E6 cells were infected with the ZIKV BeH819015;^45^ M.O.I 0.01 and incubated for 3 days to allow > 95% infection of cells. Cells were then washed, trypsinized, and resuspended in 2% FBS medium. Three hundred (300) µl of cell suspension was transferred to 8-well Lab-Tek chamber slides and incubated for 6 h or overnight prior to the experiment. The infected cells served as antigen for the detection of antibodies in sera of vaccinated mice. To determine seroconversion, antibody titrations were performed starting from 1:10 to 1:128,000 from which 100 µl was added to ZIKV-infected cells in a 96-well plate. All downstream steps were performed as described above for IFA.

### Antigens for T cell assays

For in vitro stimulation, a ZIKV E peptide pool (JPT Peptide Technologies GmbH, Berlin, Germany) and an MHC I Haplotype class-restricted YFV-17D NS3 peptide^47^ (sequence ATLTYRML, Eurogentec, Seraing, Belgium) were used. For the production of cell lysates, Vero E6 cells were infected with YFV-17D (Stamaril®, SanofiPasteur) or Zika virus (BeH819015). Two days after infection, cells were subjected to serial freeze-thaw cycles, inactivated by 254 nm UV irradiation overnight and the diluted cell lysate (50 µg/ml) was used as antigen for the stimulation of splenocytes. Non-infected Vero E6 cell lysate was used as a negative control.

### Intracellular cytokine staining and flow cytometry

Fresh mouse splenocytes were plated in a 96 round-bottom well plate (Costar, Corning Inc., Corning, NY, USA) at a density of 2 × 10^6^ cells/well and incubated overnight with either 1 µM/peptide of the ZIKV E peptide pool, or 5 µM of YFV-17D NS3 ATLTYRML or 50 µg/ml of Vero E6 cell lysate. After treatment with 5 µg/ml brefeldin A (Biolegend, San Diego, CA, USA) for 2 h, the splenocytes were stained for viability with Zombie Aqua™ (Biolegend) (1:200 dilution in PBS) for 15 min. Subsequently, extracellular markers CD3 (4 µg/ml eFluor® 450 anti-mouse CD3 antibody, ThermoFisher, Waltham, MA, USA) and CD8 (2 µg/ml APC/Cy7 anti-mouse CD8a antibody, Biolegend) were stained and cells were fixed in 2% paraformaldehyde (Sigma-Aldrich, St. Louis, MO, USA). Finally, splenocytes were permeabilized with 0.1% saponin before intracellular staining for IFN-γ (2 µg/ml APC anti-mouse IFN-γ, Biolegend), TNF-α (6.5 µg/ml PE anti-mouse TNF-α, Biolegend) and granzyme B (5 µg/ml FITC anti-human/mouse granzyme B, Biolegend). Samples were analyzed on a BD LSRFortessa™ X-20 (Becton Dickinson, Franklin Lakes, NJ, USA). Percentages of responding CD4^+^ or CD8^+^ T cells were calculated by subtracting the percentage of responders from non-stimulated samples (incubated with non-infected Vero E6 cell lysates) from corresponding stimulated samples.

### ELISPOT

An ELISPOT assay for the detection of IFN-γ-secreting mouse splenocytes was performed with a mouse IFN-γ ELISPOT kit (ImmunoSpotMIFNG-1M/5, CTL Europe GmbH, Bonn, Germany). Per well, 4 × 10^5^ mouse splenocytes were incubated with antigen (1 µM/peptide of the ZIKV E peptide pool, 5 µM of the YFV-17D NS3 ATLTYRML peptide, or 50 µg/ml of the Vero E6 cell lysates) at 37 °C for 24 h. One day after, IFN-γ spots were visualized by stepwise addition of a biotinylated detection antibody, a streptavidin-enzyme conjugate and the substrate. Spots were counted using an ImmunoSpot S6 Universal Reader (CTL Europe GmbH) and normalized by subtracting the number of spots from samples incubated with non-infected Vero E6 cell lysates from the spot number of corresponding stimulated samples.

### Measurement of pro-inflammatory cytokines and chemokines

Induction of pro-inflammatory cytokines and chemokines was analyzed in 20 μl serum using the mouse cytokine 20-plex antibody bead kit (ProcartaPlex Mouse Th1/Th2 & Chemokine Panel I [EPX200-26090-901]). Measurements of the expression of TNF-α, IFN-γ, IL-6, IL-18, CCL2 (MCP-1), CCL3 (MIP-1α), CCL4 (MIP-1β), CCL5 (RANTES), CCL7 (MCP-3), CCL11 (Eotaxin), CXCL1 (GRO-α), CXCL2 (MIP-2), CXCL10 (IP-10), GM-CSF, IL-1β, IL12p70, IL-13, IL-2, IL-4, and IL-5. Measurements were performed using a Luminex 100 instrument (Luminex Corp., Austin, TX, USA) and were analyzed using a standard curve for each molecule (ProcartaPlex). Data are displayed as median values. Statistical analysis was performed using a Mann–Whitney two-tailed test

### Statistical analysis

Data were analyzed using GraphPad Prism v7 software and expressed as mean values ± standard error of mean (SEM). Comparison of groups was performed using either one-way ANOVA or Mann–Whitney two-tailed test, with *P* values < 0.05 indicating a statistically significant difference between groups.

For vertical transmission studies and fetal RNA quantifications, GraphPad Prism software (version 6.02) was used for the analyses, where statistical tests were applied according to the distribution, variance, normality of each dataset as well as the number of biological replicates in each experiment as specified in the figure legends. The Iglewicz and Hoaglin’s Test was applied to exclude outliers, where the modified *Z*-score was set to 3.5. Kolmogorov–Smirnov test was used to determine distribution of dataset and normality. Multiple comparisons were performed with Kruskal–Wallis followed by Dunn’s post hoc tests for nonparametric datasets.

Levels of significance: ****P *<* 0.0001, ***P *<* 0.001, **P *<* 0.01, *P *<* 0.05 for all statistics herein; ns = not significant.

## Supplementary information

Supplementary Figures and Tables

## Data Availability

The authors declare that all data supporting the findings of this study are available within the paper and supplementary files.

## Supplementary information

**Supplementary information** accompanies the paper on the *npj Vaccines* website (10.1038/s41541-018-0092-2).
