# Supplementary material for: A yellow fever–Zika chimeric virus vaccine candidate protects against Zika infection and congenital malformations in mice
Source: NPJ Vaccines. 2018 Dec 13;3:56. doi: 10.1038/s41541-018-0092-2 (PMC6292895; doi:10.1038/s41541-018-0092-2)
Supplement: Supplementary file 1 — Supplementary Figures and Tables [file 41541_2018_92_MOESM1_ESM.pdf]

Polyprotein	***C protein*** *(carboxy-terminus)* ***C anchor domain*** ***prM protein*** *(amino-terminus)*	
**YFV-17D**	…LSSRKRR-(2b/3)-SHD**VLTVQFLILGMLLMTGG-(sign)-VTL…	**functional**
**JEV SA14-14-2**	…GRKQNKR-(2b/3)-GGNEGSIMWLASLAVVIACAGA-(sign)-MKL…	**functional**
**ZIKV-Yap2007**	…RKEKKRR-(2b/3)-GTDTSVGI****VGLLLTTAMA-(sign)-VEV…	**functional**
**Chimerivax-JE**	…LSSRKRR-(2b/3)-SHD**VLTVQFLILGMLLMTGG-(sign)-MKL…	**functional**
**Chimeric YF/ZIKV_ZIK-Canch**	…LSSRKRR-(2b/3)-GTDTSVGI****VGLLLTTAMA-(sign)-VEV…	**functional**
**Chimeric YF/ZIKV_YF-Canch**	…LSSRKRR-(2b/3)-SHD**VLTVQFLILGMLLMTGG-(sign)-VEV…	**non-functional**

Supplementary Figure 1. Structure of C-prM junctions in wild-type and chimeric flaviviruses used in the present study. Amino acid sequences upstream and downstream of the C anchor domain (= prM signal peptide) are shown in single letter SI abbreviation; (2b/3) – NS2b/3 protease cleavage site; (sign) – signal peptidase cleavage site; * – gap introduced to facilitate sequence alignments.

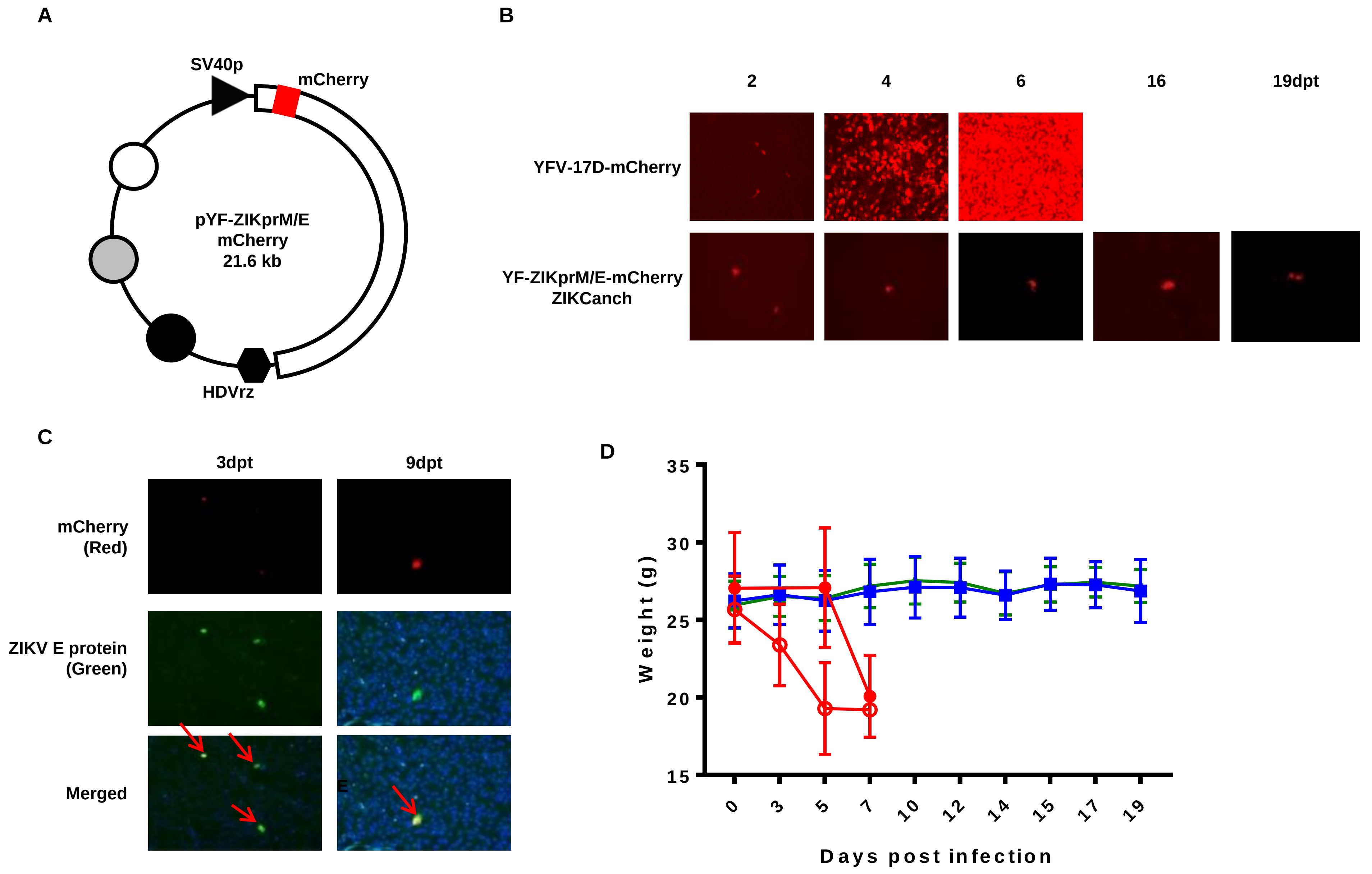

E

Dose	Dead/Total number of mice	% Mortality	MDE ± SEM
10^4 PFU	13/13	100 %	12,3 ± 2,2
10^3 PFU	19/19	100 %	13,2 ± 2,2
10^1 PFU	3/3	100 %	13,3 ± 3,1
10^0 PFU	10/17	59 %	14,7 ± 2,6
10^{-1} PFU	4/7	58%	15,8 ± 2,1

Supplementary Figure 2. Infectivity of mCherry YF-ZIKprM/E-ZIKCanch chimeric virus. (A) Based on the YFV-17D, YF-ZIKprM/E chimeric viruses were constructed that are tagged with an mCherry gene that is expressed as N-terminal translational fusion to the YFV-17D polyprotein essentially as described by Fischl & Bartenschlager (48). As a comparator, YFV-17D carrying an mCherry reporter was constructed using the same backbone. (B) Vero E6 cells were transfected with pYFV-17D-mCherry (upper panel) or pYF-ZIKprM/E-mCherry (lower panel) and monitored for virus propagation. (C) Staining of Vero E6 cells with mAb 4G2, 3 and 9 days post pYF-ZIKprM/E-mCherry transfection to assess antigenic expression. Co-localization of mCherry and envelope protein staining with 4G2 is indicated with arrows, The signals stay confined to a few cells and are not spreading during the period of inspection. (D) Weight change over time of AG129 mice inoculated intracranially with YF-ZIKprM/E viruses encoding either a YFV anchor (green triangles) or ZIKV anchor (blue squares). Infection outcomes of the intracranial inoculation of adult wild-type BALB/c (red, closed circles) and ifnar$^{-/-}$ (red, open circles) with each 1x10^3 PFU of YFV-17D are shown for comparison. Data are presented as mean values with error bars indicating SEM. Each group represents n = 3 – 5 biologically independent samples. (E) Determination of a minimum lethal dose of parental YFV-17D in AG129 mice. Groups of adult AG129 mice were infected i.p. with different doses of YFV-17D and observed for mortality within 21 days post infection. Results are presented as mean ± SEM. MDE – mean days to euthanasia. Dpt – days post transfection

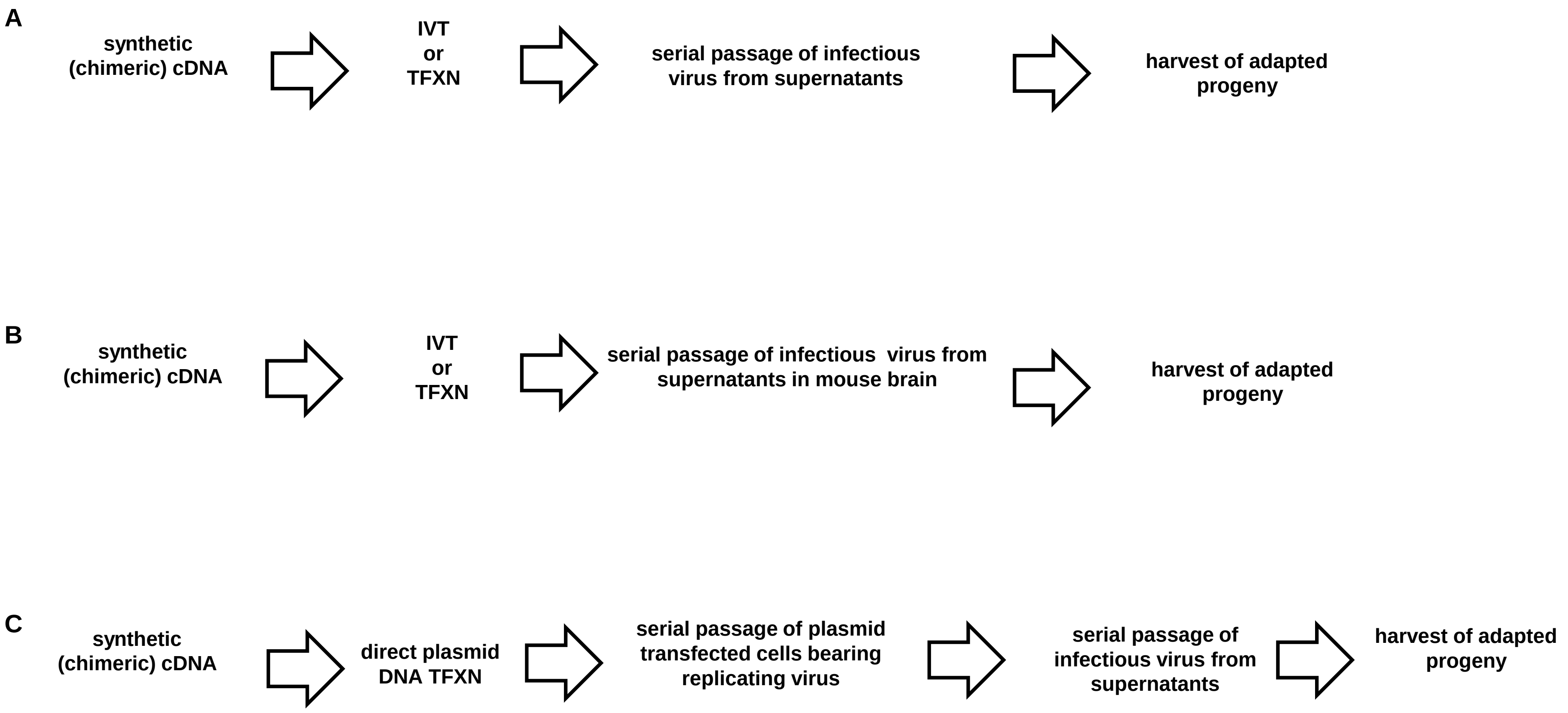

Supplementary Figure 3. Propagation and growth of flaviviruses. Comparison of classical strategy to (A) generate and adapt recombinant flaviviruses (e.g. Arroyo et al. 2001 [6]) with our novel staged approach (C). Alternative to (A), infectious progeny has been amplified by intracranial inoculation into the brains of mouse pups (B). cDNA – copy DNA of viral RNA genome; IVT – in vitro transcription; TFXN – transfection.

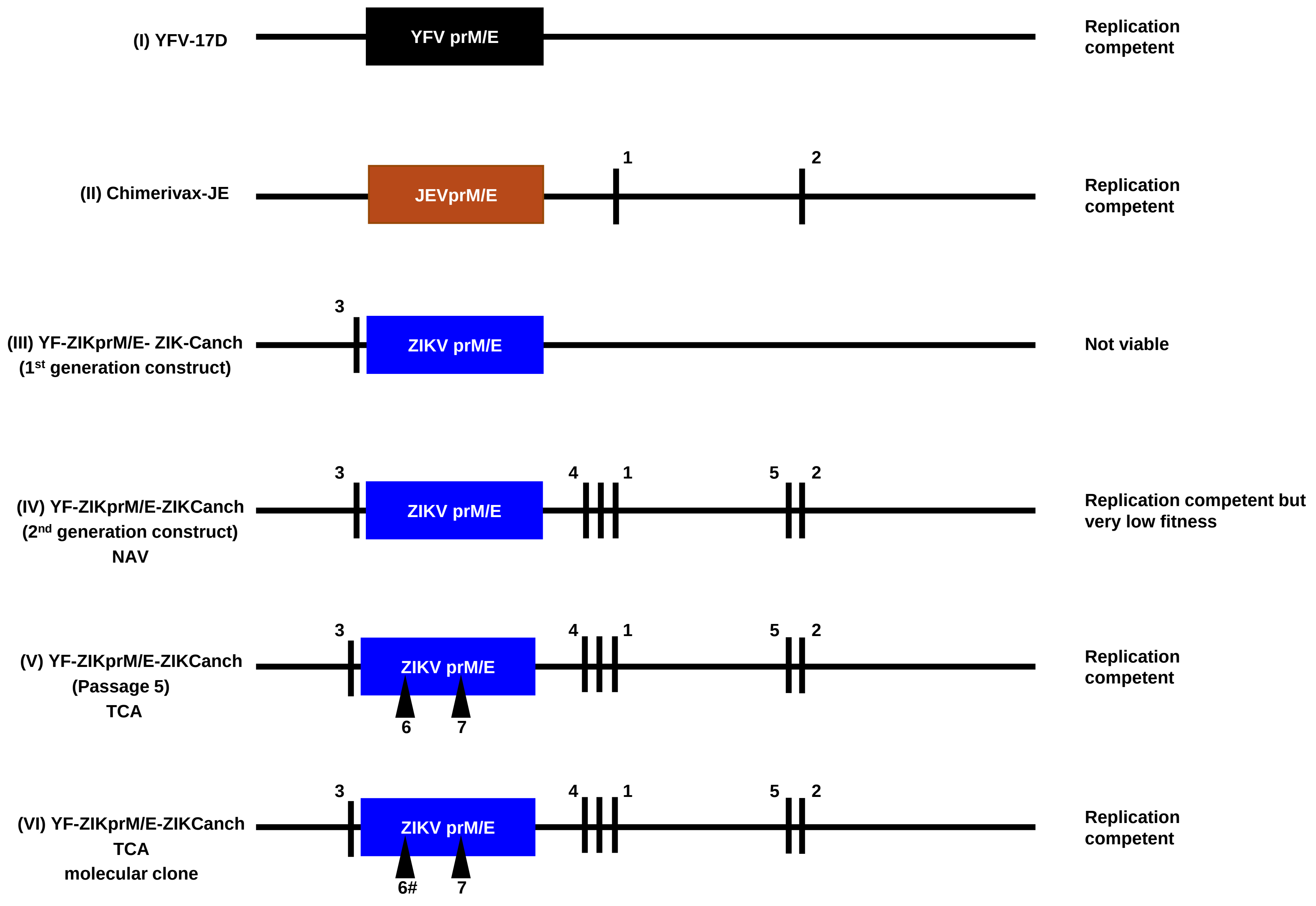

6: Heterogeneous A (majority) and G (wild-type/minority)
6#: Majority A cloned into molecular clone of tissue culture adapted virus

NAV – non adapted virus; TCA – tissue culture adapted

Supplementary Figure 4. Mutational pattern of synthetic chimeric flaviviruses: Chimerivax-JE and novel YF/ZIKprM/E-ZIKCanch. The copy DNAs of the parental virus YFV-17D (I) had been modified to carry the prM/E sequence of the JE SA14-14-2 vaccine (6). Respective Chimerivax-JE virus (II) required adaptive mutations 1 and 2 for full replication fitness. Construct (III) that is based on YFV-17D [as described in PCT/EP2014/058459 (WO2014174078)] with the capsid signal peptide and prM/E sequences of the ZIKV-Yap 2007 strain plus an additionally engineered (translationally silent) mutation 3 (*Xho1* restriction site) was not viable. Introduction of mutations 1 (accompanied by a translationally silent *BstE2* restriction site; double nucleotide mutation 4) and 2 (accompanied by a translationally silent *Nhe1* restriction site; mutation 5) into the backbone of (III) yield a viable YF-ZIKprM/E chimera, yet with poor replication fitness and failure to produce viable infectious progeny (NAV – non-adapted virus). Intracellular and extracellular passaging leads to fixation of mutations 6 and 7 (triangles) in passage 3 to 5 (recombinant virus (V); TCA – tissue culture-adapted virus). Mutations G1097A (mutation 6) and C2343T (mutation 7) were re-introduced in (IV) by site-directed mutagenesis to generate a molecular clone of the TCA virus (VI).

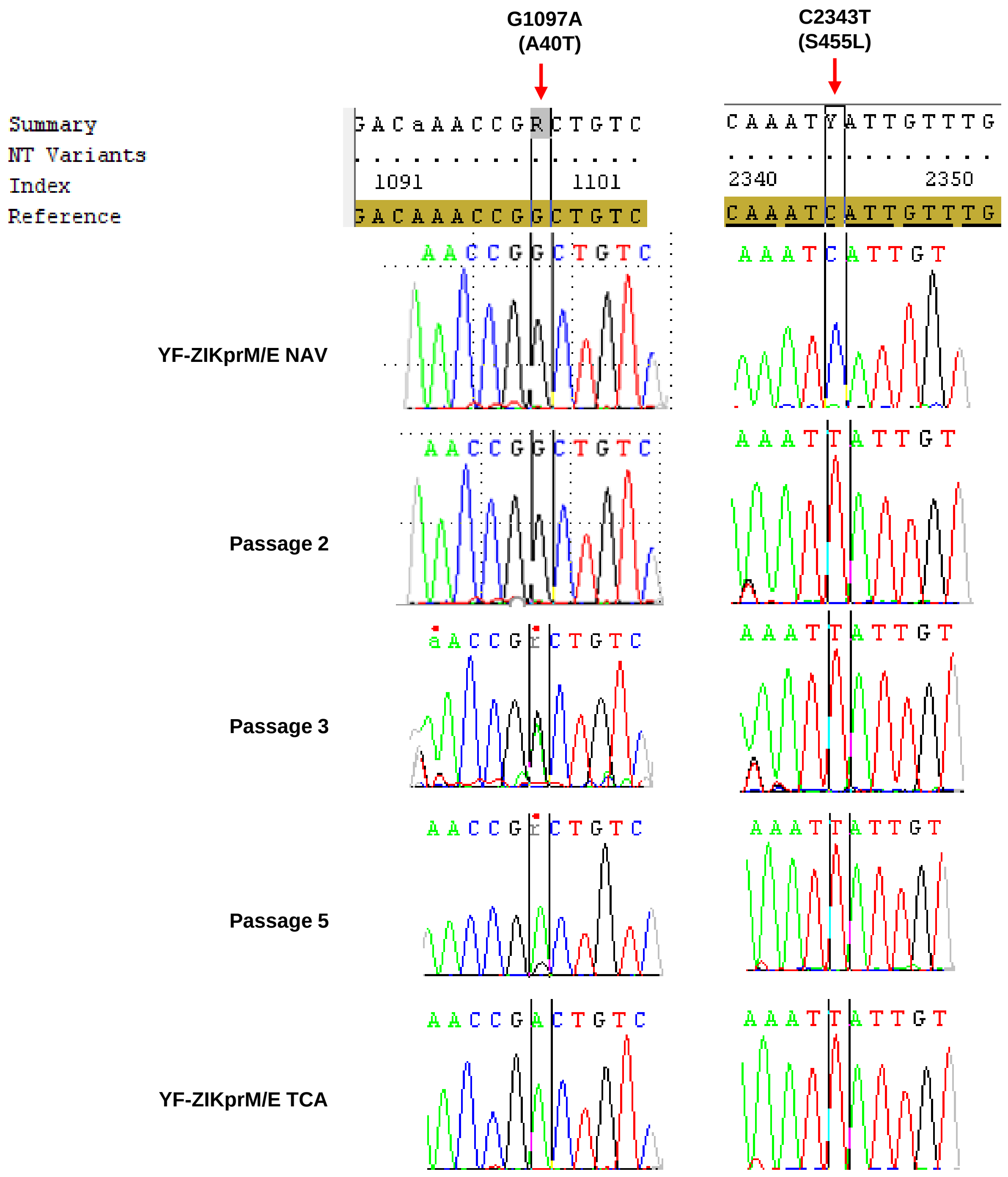

Supplementary Figure 5. Evolution of tissue culture-adapted (TCA) mutation from non-adapted virus (NAV) and reverse engineering of TCA mutation into YF-ZIKprM/E. Chromatograms from Sanger sequencing reactions of RT-PCR amplicons from subsequent tissue culture supernatants. Red arrows point to the mutations G1097A and C2343T corresponding to amino acid changes A40T and S455L in the ZIKV E protein, respectively, and their successive evolution. Sequencing tracks for the molecular cDNA clones of the NAV and TCA viruses serve as controls.

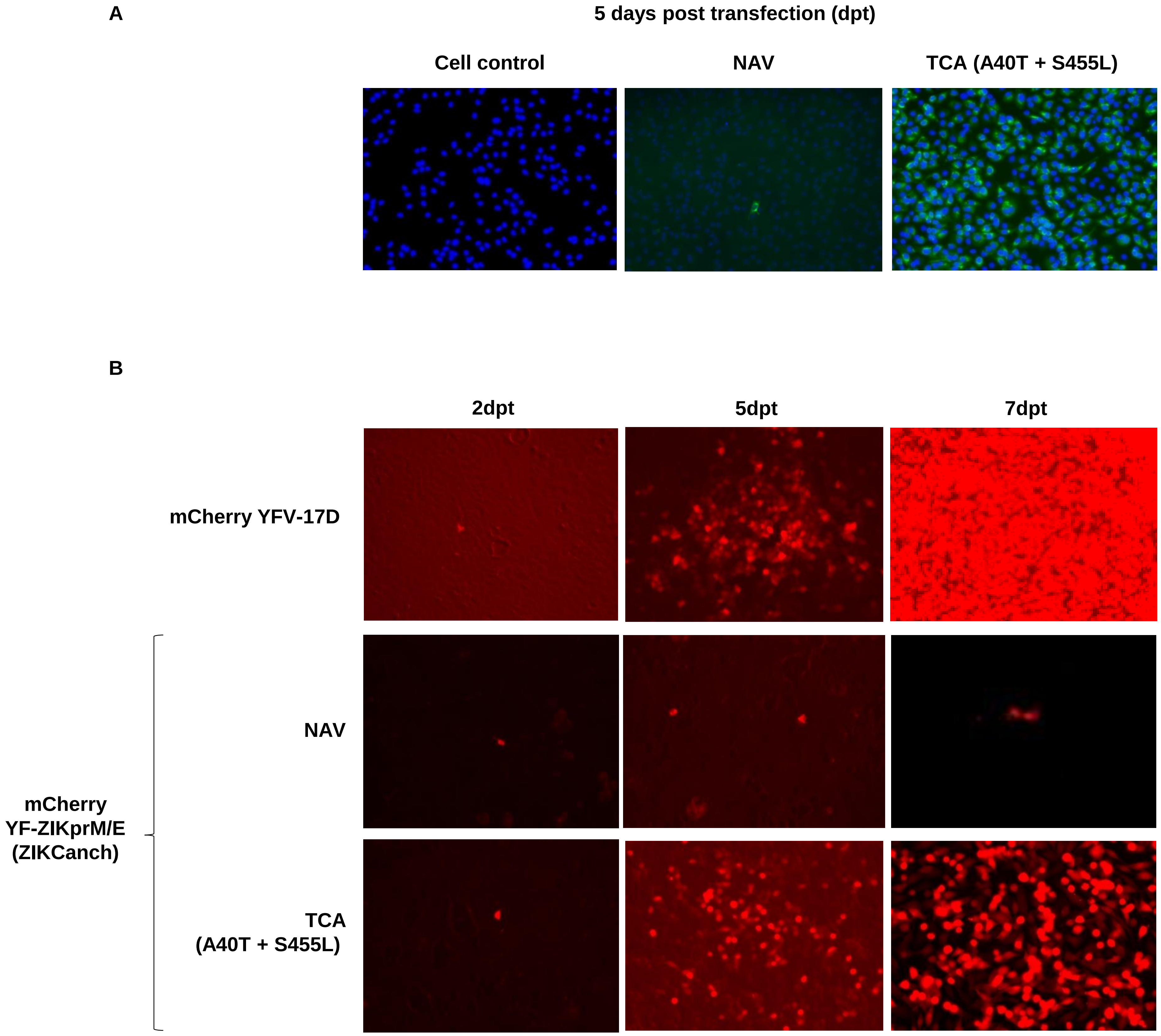

Supplementary Figure 6. Reverse engineering of tissue culture-adaptive (TCA) mutations into (A) YF-ZIKprM/E and (B) mCherry-tagged YF-ZIKprM/E, and propagation of the resulting viruses in Vero E6 cells. (A) Clonal non-adapted virus (NAV) and TCA viruses were generated by tranfection of Vero E6 cells with 2.5 µg of plasmid. Infection was detected by staining for ZIKV E proteins with mAb 4G2 5 days post transfection (after splitting cells and seeding in 8-chamber slides). (B) Plasmids encoding for mCherry-tagged YF-ZIKprM/E variants of either NAV and a molecular clone of TCA virus (A40T + S455L) were transfected in Vero E6 cells, and the replication and spread of viral progeny in tissue culture was monitored by fluorescence microscopy. A mCherry tagged clone of YFV-17D served as viable and readily propagating positive control.

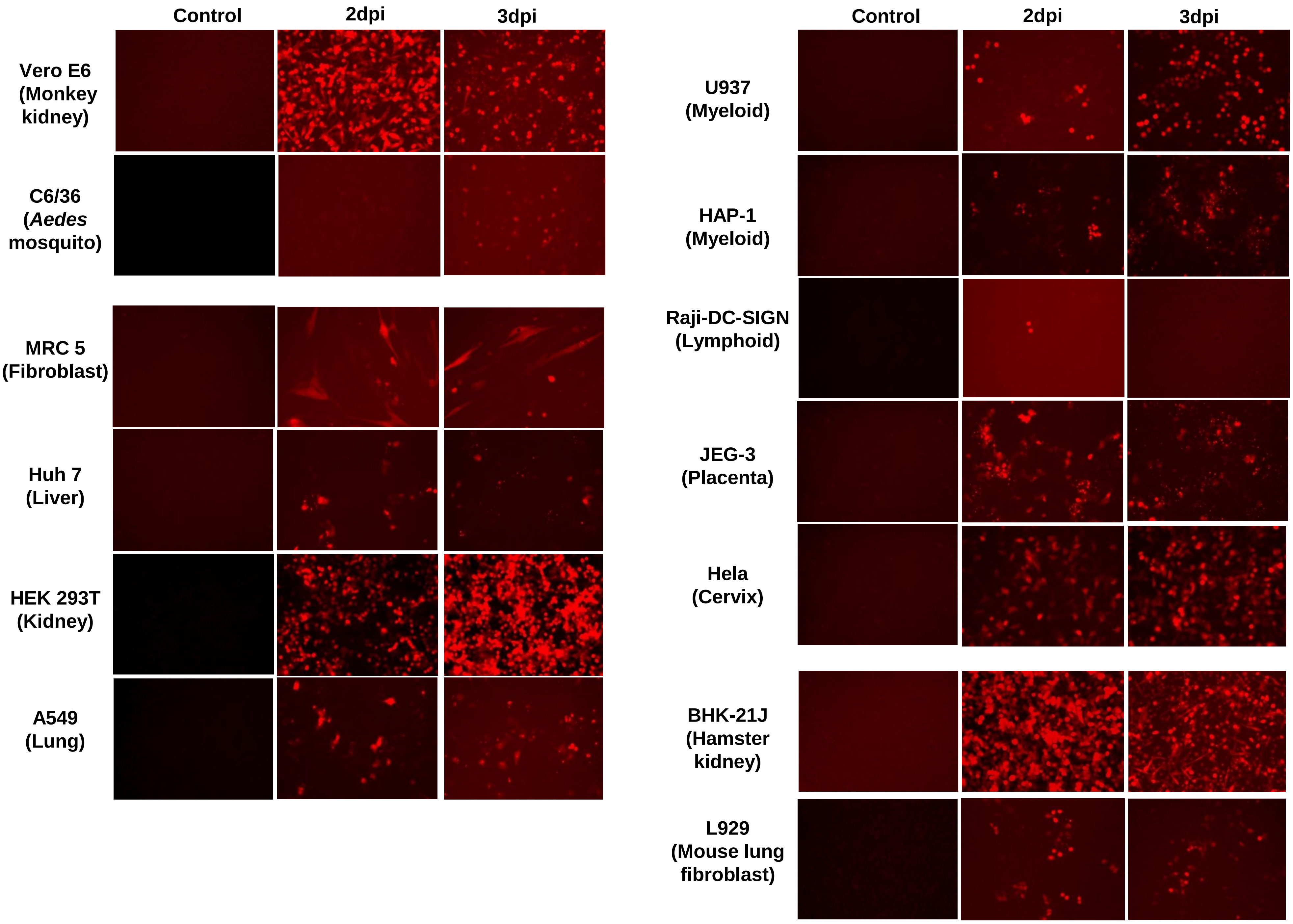

Supplementary Figure 7. Infectivity of TCA YF-ZIKprM/E in different cell lines. Cells of different species (monkey, mosquito, human, hamster and mouse) and tissue origin (fibroblast, liver, kidney, lung, blood/bone marrow, placenta and cervix) were seeded in 96-well plates and infected with TCA mCherry-tagged YF-ZIKprM/E at a MOI of 1. Pictures were taken at days 2 and 5 post infection using the Floid Cell Imaging Station, ThermoFisher, Germany. The tissue origin of the cell lines used was assigned according to the classification provided by the Human Protein Atlas (https://www.proteinatlas.org/) where applicable.

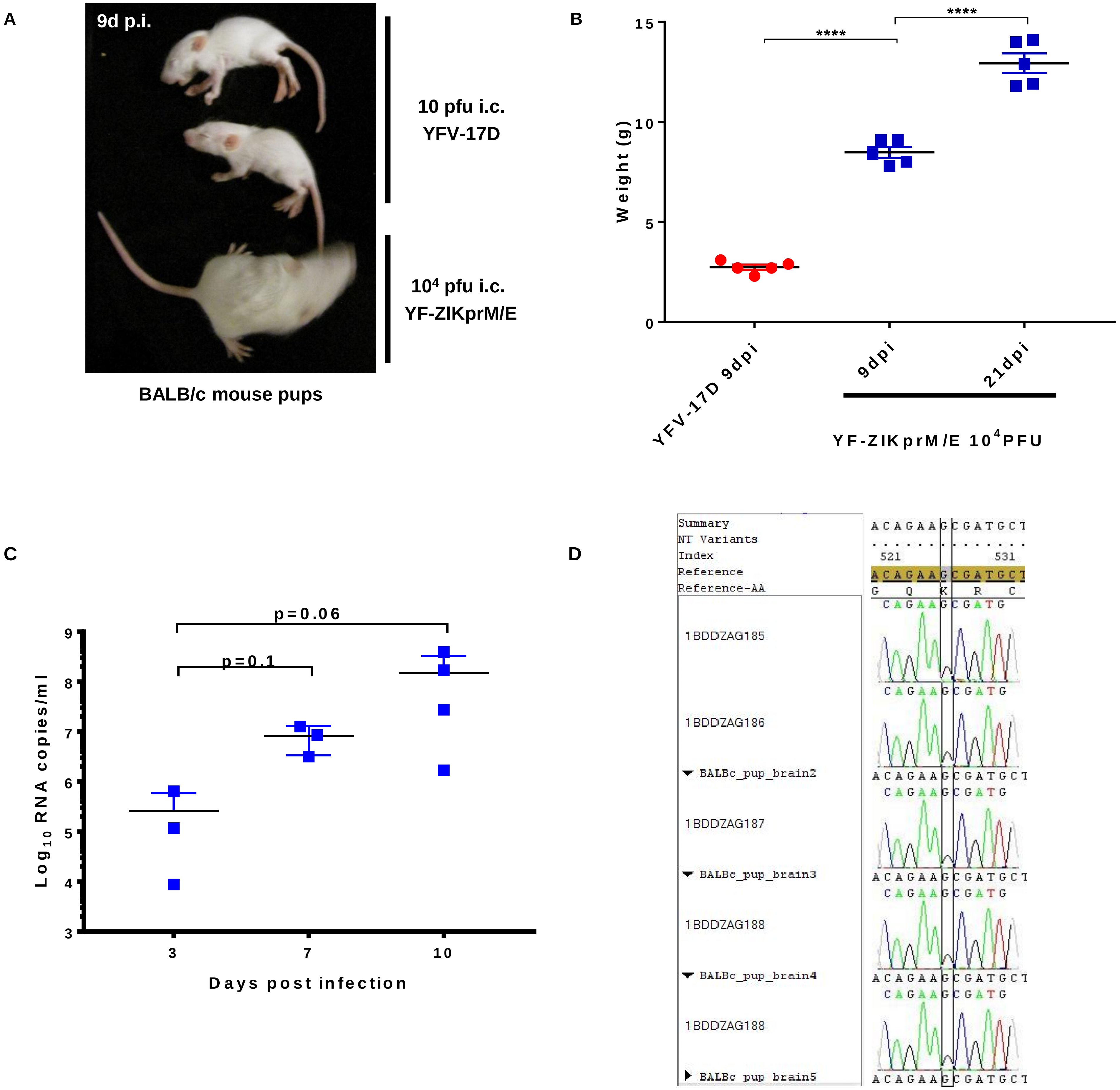

Supplementary Figure 8. Safety of YF-ZIKprM/E and YFV-17D in 5 days old BALB/c pups. (A) Caption of mice 9 days after intracranial (i.c.) inoculation with either 10 PFU of YFV-17D (upper panel) or $1x10^4$ PFU of YF-ZIKprM/E (lower). YFV-17D-inoculated pups show growth retardation, apathy and paralysis. YF-ZIKprM/E-inoculated mice are growing as expected for unaffected mice and are actively moving. (B) Weights of BALB/c pups after intracranial inoculation with either 10 PFU of YFV-17D (red circles, n=5) and $1x10^4$ PFU of YF-ZIKprM/E (blue squares, n=5). Data are presented as mean ± SEM. Mann-Whitney two-tailed test was used for statistical analysis, ****P<0.0001. (C) Detection of viral in brains following i.c. inoculation of $1x10^4$ PFU of YF-ZIKprM/E into n=10 BALB/c pups. At 3, 7 and 10 dpi, three, three and four animals were sacrificed, respectively, and RNA was extracted from their brains for detection of viral genomes by qRT-PCR. (D) Absence of S135N mutations. Chromatograms from Sanger sequencing reactions of RT-PCR amplicons obtained from four brains harvested 10 dpi. Nucleotide G525 from codon 139 [AGC > serine, mutated to AAC > asparagine in epidemic strains (18)], indicated by box. Codon 139 in the ZIKV polyprotein corresponds to prM amino acid 17. Sequencing tracks for the inoculum virus (upper panel) serves as controls. Data are presented as mean values with error bars indicating SEM from 2 independent experiments, n=2 or 3 per group.

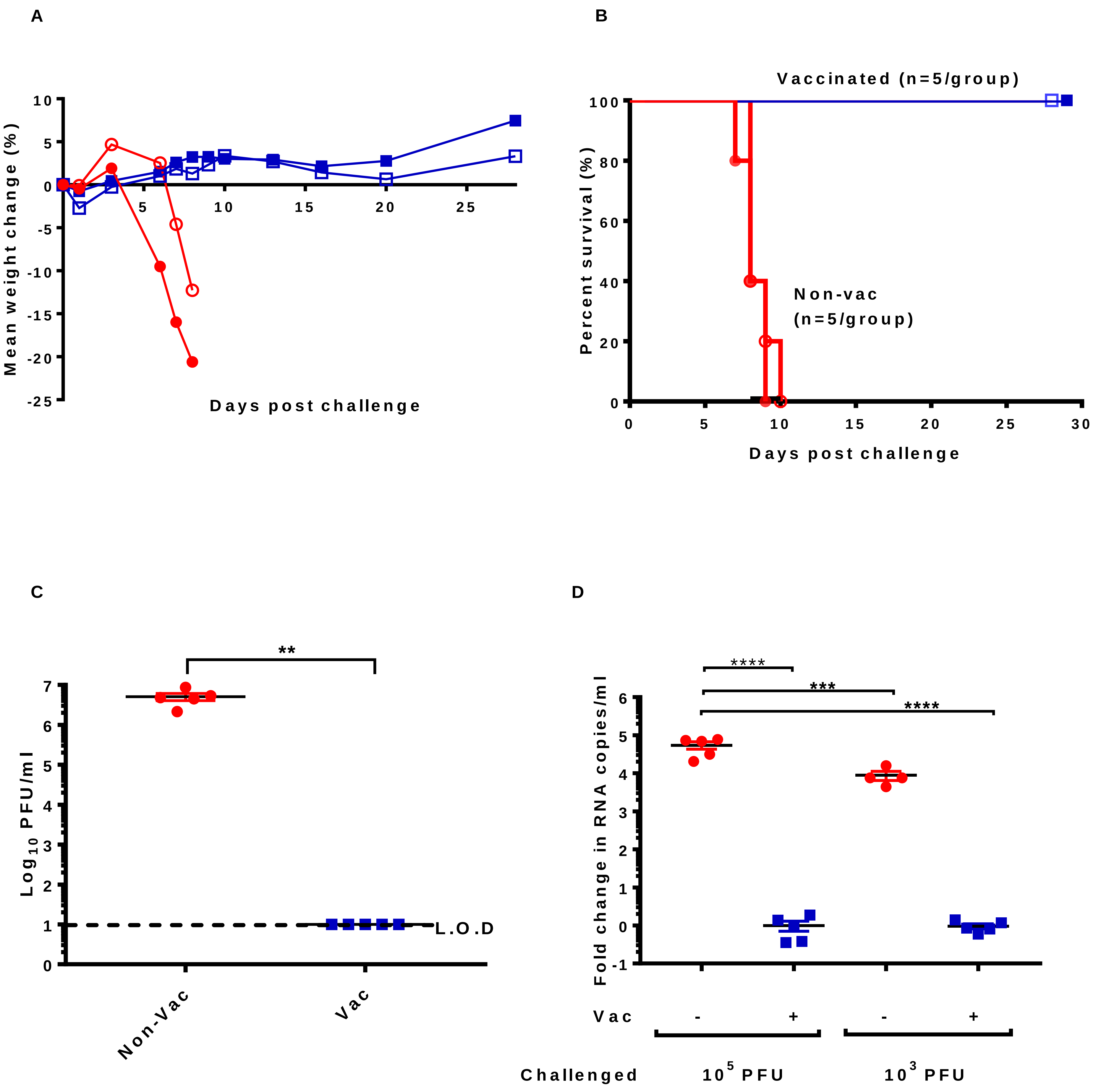

Supplementary Figure 9. Seroconversion and survival of mice vaccinated with YF-ZIKprM/E. Mean weight change (A) and survival (B) of vaccinated (blue line) and sham-vaccinated (red line) AG129 mice following i.p. challenge with 1x10^3 PFU (open circle/square) or 1x10^5 PFU (closed circle/square) of ZIKV SL1602 (n=5/group). (C) Serum virus loads presented in PFU/mL at 3 days post challenge in AG129 mice that had either been vaccinated with 1x10^4 PFU of YF-ZIKprM/E (blue squares, n=5) or that were sham-vaccinated (red circles, n=5) and challenged with 1x10^5 PFU ZIKV SL1602. Dotted lines denote the limit of detection of the assay. (D) Serum virus loads at 3 days post-challenge in AG129 mice that were vaccinated with 1x10^4 PFU of YF-ZIKprM/E (blue squares, n=5/group) and were challenged with 1x10^5 PFU or 1x10^3 PFU of ZIKV SL1602. Data are presented as mean values with error bars indicating SEM from biologically independent samples. Data are represented as mean ± SEM. Mann-Whitney two-tailed test was used for statistical analyses. Asterisks indicate a significant difference: *P-values <0.05, **P-values <0.01, ***P-values <0.001, ****P-values <0.0001. Background (dotted line) represents average RNA copies of vaccinated mice before challenge. Horizontal dotted lines denote the limit of detection (L.O.D.) of the assay.

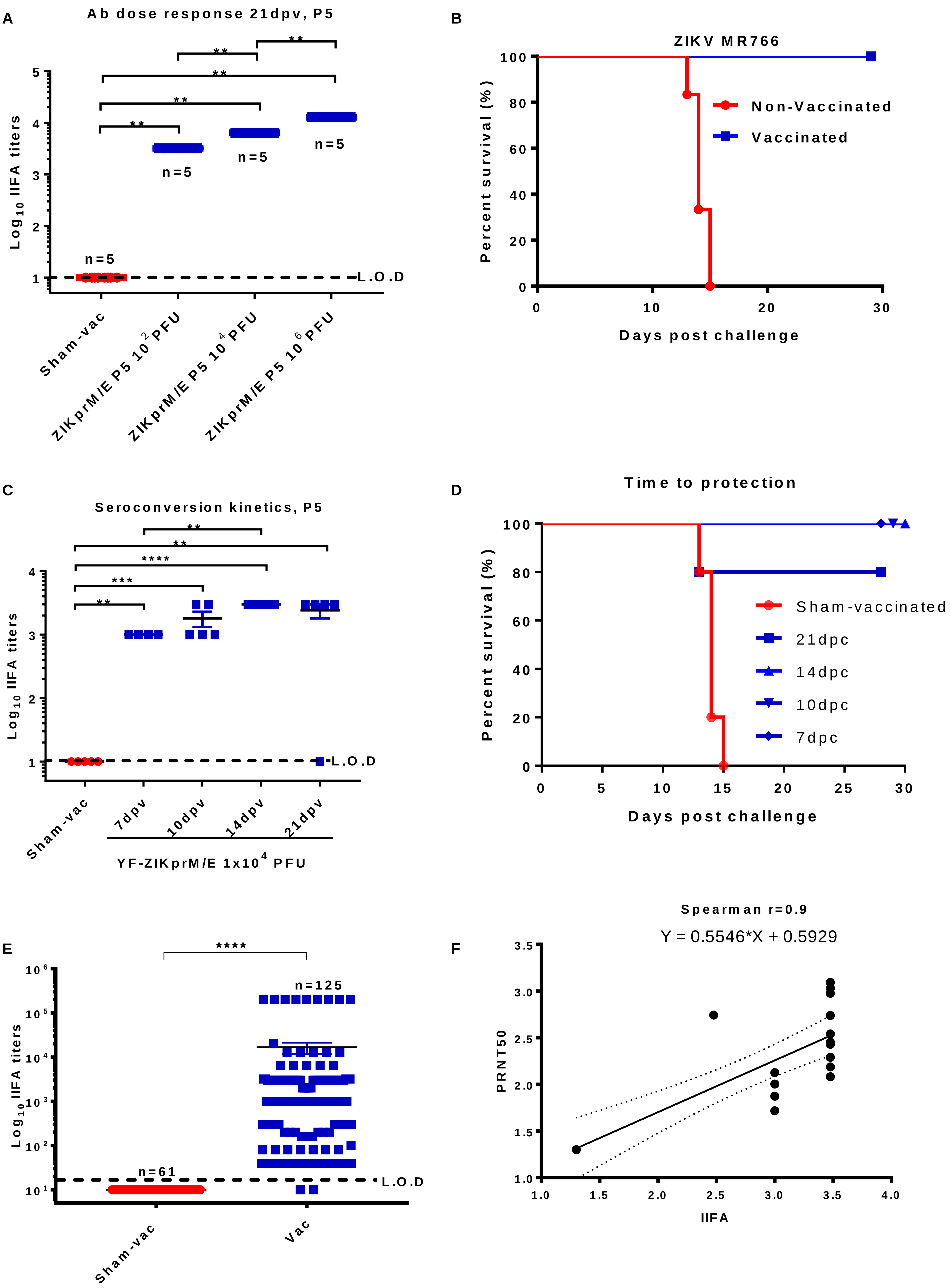

Supplementary Figure 10

Supplementary Figure 10. YF-ZIKprM/E is immunogenic and fully protective at low vaccine doses and confers a benefit early after vaccination. (A) AG129 mice (n=5/group) were vaccinated with $1x10^2$, $1x10^4$ and $1x10^6$ PFU of YF-ZIKprM/E, respectively. After 21 days, ZIKV-specific Ab responses were measured by IIFA. (B) Survival (days to euthanasia) of low dose vaccinated and sham-vaccinated mice. Mice vaccinated with $1x10^2$ PFU of YF/ZIKprM/E (blue squares) and sham-vaccinated (red circles) mice (n=6/group) were challenged 21 dpv with $1x10^4$ PFU of ZIKV MR766. (C) Kinetics of seroconversion to ZIKV-specific Abs following YF-ZIKprM/E vaccination. Mice were vaccinated with $1x10^4$ PFU of YF-ZIKprM/E (blue squares, n=5/group) or sham-vaccinated (closed circles, n=5/group), at various time points prior to challenge (D). Blood was drawn immediately before i.p. challenge with $1x10^4$ PFU of ZIKV MR766 and analysed by IIFA. (D) Survival curve of sham-vaccinated mice (closed circles, n=5/group) and mice vaccinated (blue symbols, n=5/group) at various time points prior to challenge. Dpc= days pre-challenge, dpv= days post-vaccination. Data are presented as mean ± SEM. Mann-Whitney test was used for statistical analyses. Asterisk indicates a significant difference **P-values<0.01, ***P-values <0.001, ****P-values <0.0001. (E) Seroconversion status of all mice used in this study regardless of dose regimen, mouse strain, time of vaccination. Data are presented as mean ± SEM. Mann-Whitney two-tailed test was used for statistical analysis, ****P<0.0001. Overall seroconversion rate was 123/125 > 98%. (F) Correlation of PRNT50 and IIFA endpoint titers corresponding to data presented in Fig 2A (n=20/group). Cut-off values for both assays was set at a serum dilution 1:20, resulting in consistent detection of seroconversion to ZIKV-specific Ab and quantitative assessment of Ab titers by either method. Data are presented as mean values with error bars indicating SEM from biologically independent samples. Mann-Whitney two-tailed test was used for statistical analyses. Asterisk indicates a significant difference **P-values<0.01, ***P-values <0.001, ****P-values <0.0001. Horizontal dotted lines denote the limit of detection (L.O.D.) of the assay.

A

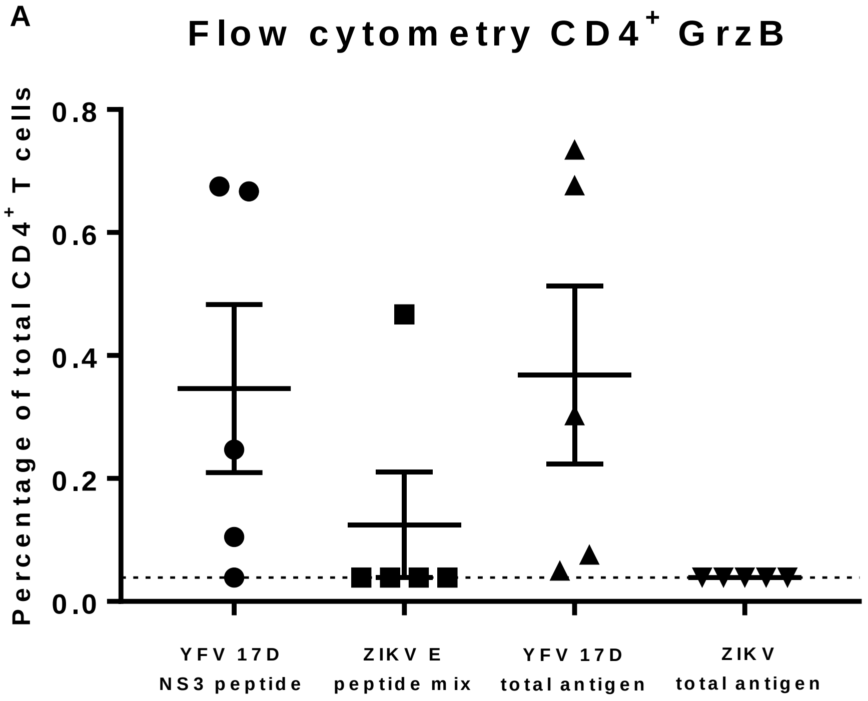

B

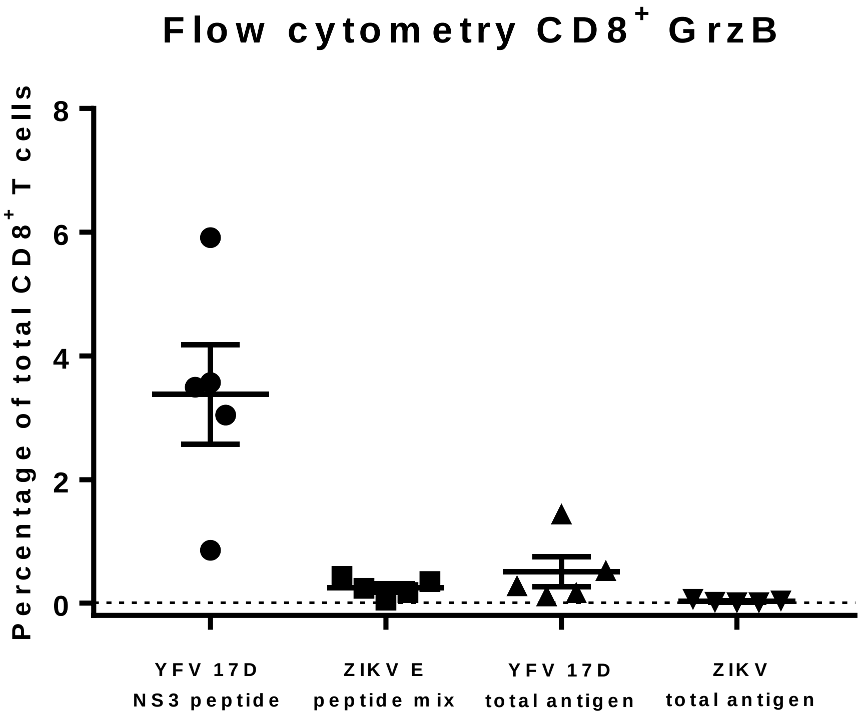

Supplementary Figure 11. Cytotoxic T cell responses after vaccination of mice with YF-ZIKprM/E. Intracellular cytokine and granzyme B (GrzB), staining to assess (A) $CD4^+$ and (B) $CD8^+$ responses after overnight stimulation with indicated antigens. Data are presented as mean values with error bars indicating SEM from 2 independent experiments, n=2 or 3 per group. Data are presented as mean ± SEM. Background (dotted line) represents average values for sham-vaccinated mice.

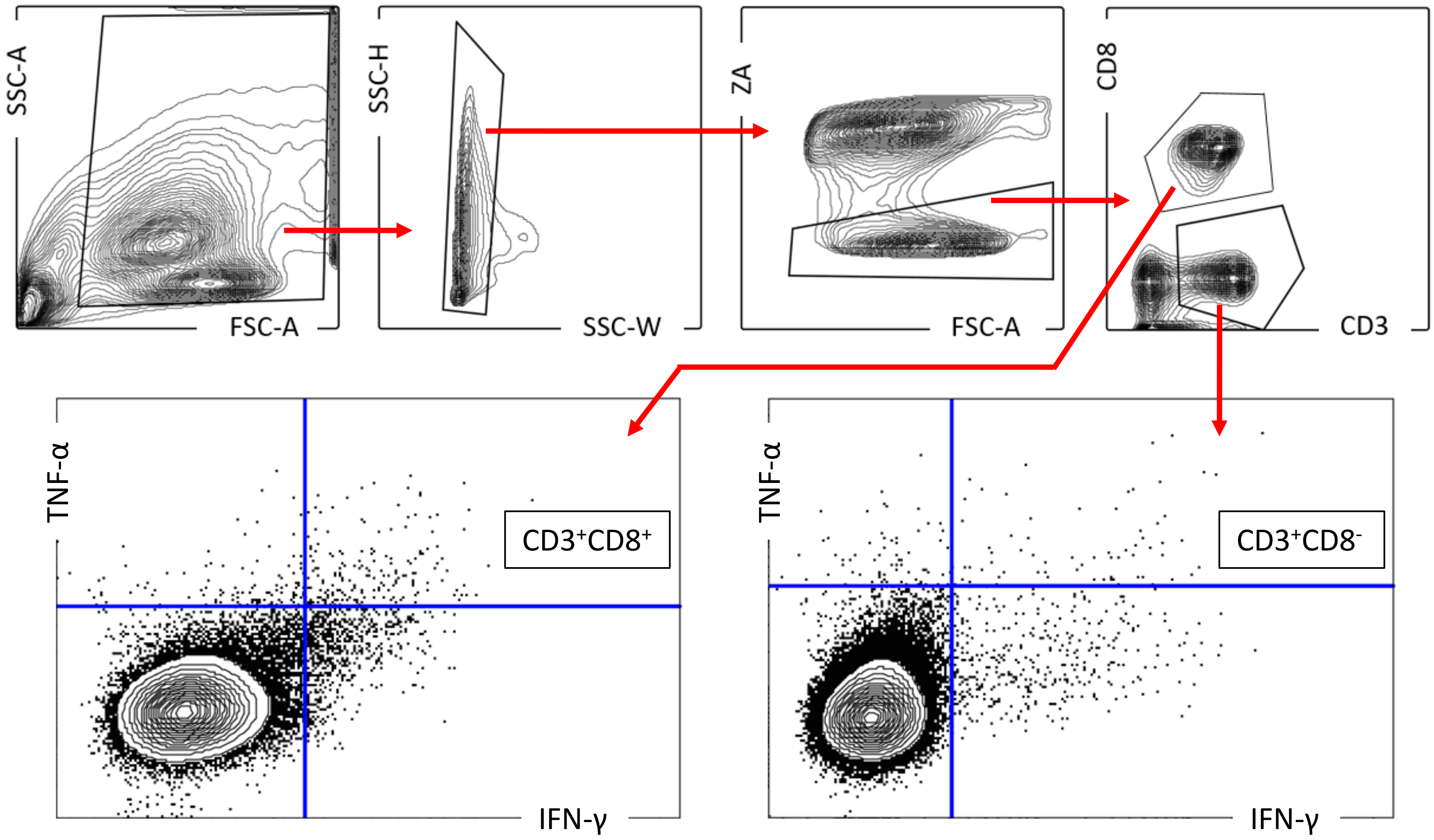

Supplementary Figure 12. Gating strategy for intracellular cytokine staining. In a first step, debris was eliminated by gating out the FSC-low population in a FSC-A vs SSC-A plot. Then, in a SSC-W vs SSC-H plot, doublets were excluded from further analysis by gating out high SSC-W events. Dead cells were gated out by selecting only the Zombie Aqua (ZA) negative population in a FSC-A vs ZA plot. Finally, $CD8^+$ and $CD4^+$ T cells are gated based on positivity for CD3e (eFluor450-conjugated), and positivity (CD8) or negativity (CD4) for CD8a (APC/Cy7-conjugated). Samples from non-vaccinated negative control mice were used to define boundaries between positive and negative staining cell populations. Arrows indicate the various steps during gating.

Supplementary Table 1: Workflow for the propagation of highly attenuated chimeric YF-ZIKprM/E

		Protocol for the propagation of the highly attenuated YF-ZIKprM/E	
	Day -1	Seed Vero E6 cells at 5 x 10^6 cells/well in 6-well plates	
Transfection	Day 0	Transfect cells with 2.5µg of plasmid	
	Day 1		
Intracellular passage #1	Day 2	Wash/Trypsinize and transfer all cells to 25 cm^2 flask + 2ml supernatant	
	Day 3	1 day post split (dps)	IFA [1]
	Day 4	2 dps	
	Day 5	3 dps	IFA [1]
	Day 6	4 dps	
	Day 7	5 dps	IFA [1]
Intracellular passage #2	Day 8	Wash/Trypsinize and transfer all cells to 75 cm^2 flask + 3ml supernatant	
	Day 9	1 dps	IFA [2]
	Day 10	2 dps	
	Day 11	3 dps	IFA [2]
	Day 12	4 dps	
	Day 13	5 dps	IFA [2]
Intracellular passage #3	Day 14	Wash/Trypsinize and transfer all cells to 150 cm^2 flask	
	Day 15	1 dps	IFA [3]
	Day 16	2 dps	
	Day 17	3 dps	IFA
	Day 18	4 dps	
	Day 19	5 dps	IFA
Intracellular passage #4	Day 20	Wash/Trypsinize and transfer 1/3 cells to 150 cm^2 flask + 1/3 fresh cells	
First (extracellular) passage of supernatant	Day 21	1 dpi	IFA [3]
	Day 22	2 dpi (Infection of naïve Vero cells in chamber slides with 200 µl of cell supernatant)	
	Day 23	3 dpi	IFA [3]
	Day 24	4 dpi	
	Day 25	5 dpi	IFA [3]

Immunofluorescence Assay (IFA) was used at indicated time points to monitor the infectivity and spread of the virus. [1], [2], [3]: results depicted in Fig 1B, panel 1, 2 and 3, respectively.

Supplementary Table 2: Unique engineered and acquired mutations during passaging of YF-ZIKprM/E

Virus [construct in Suppl Fig. 4] (nucleotide position in YFV)	409	1097*	2343*	3979	3985	4025	7288	7319
Mutation # in suppl fig 2	3	6	7	4	4	1	5	2
YFV-17D [I] (WO2014174078)	A			T	T	G	T	G
Chimerivax-JE [II] (Arroyo et al. 2001)	A			T	T	A	A	A
YF/ZIKprME [IV] (molecular cDNA construct (NAV))	G	G	C	G	C	A	A	A
YF/ZIKprME passage 5 (TCA) [V]	G	R (A,G)#	T	G	C	A	A	A
Amino acid change	-	Ala>Thr	Ser>Leu	-	-	Val>Met	-	Glu>Lys
Genomic region	C	E	E	NS2A	NS2A	NS2A	NS4A	NS4B

* – YF-ZIKprME-ZIKCanch numbering; # – majority A; NAV – non-adapted virus; TCA – tissue culture-adapted

Supplementary Table 3: Heat map of cytokines measured 3 days after ZIKV SL1602 challenge

	GM-CSF (42)	IFN-gamma (38)	IL-1 beta (19)	IL12p70 (39)	IL-13 (35)	IL-18 (66)	IL-2 (20)	IL-4 (26)	IL-5 (27)	IL-6 (28)
Non-vaccinated	0,05 ± 0,05	272,4± 18,2	0,0	0,13±0,3	0,0	87,7±6,2	0,0	0,0	0,2±0,2	2,5±1,3
Vaccinated 1x10^6 PFU	0,0	61,1±10,8	0,0	0,02±0,02	0,0	6,7±4,6	0,0	0,0	2,9±0,7	1,7±2,1
Vaccinated 1x10^{4}PFU	0,0	22,4±4,8	0,0	0,0	0,0	0,0	0,0	0,0	0,4±0,4	0,8±0,8
Vaccinated 1x10^{2}PFU	0,0	10,6±2,3	0,0	0,0	0,0	0,0	0,0	0,0	0,9±0,9	0,0
	TNF-alpha (45)	Eotaxin (62)	GRO-alpha (43)	IP-10 (22)	MCP-1 (51)	MCP-3 (48)	MP-1alpha (47)	MP-1beta (72)	MP-2 (55)	RANTES (44)
Non-vaccinated	2,1±2,1	30,0±8,3	14,9±4,1	9,4±1,0	106±12,2	52,8±7,3	0,01±0,004	0,6±0,4	6,0±1,7	0,0
Vaccinated 1x10^6 PFU	0,3±0,3	61,5±6,8	6,7 ±2,7	5,4±0,6	11,7±4	21,5±1,1	0,0	0,02±0,06	8,7±2,2	0,0
Vaccinated 1x10^4 PFU	0,0	26,7±3,2	2,7±0,6	1,3±0,5	3,0±3	15,2±2,3	0,0	0,0	3,8±0,6	0,0
Vaccinated 1x10^2 PFU	0,0	30,2±7,2	5,8±2,0	5,3±4,0	4,2±2,6	14,1±1,3	0,0	0,0	4,9±1,4	0,0

Induction of pro-inflammatory chemokines and cytokines was measured 3 days after infection of vaccinated (n=5) and sham-vaccinated (n=5) mice. Values displayed are averages of each cytokine measurement per group, represented as mean ± SEM. Colors indicate relative increase or decrease in cytokine expression following ZIKV SL1602 challenge within each group relative to sham-vaccinated animals.

Supplementary Table 4: List of all primers used in this study

Name	Sense	Sequence
pShuttle(+) seq	Forward	TAATAAGCGGATGAATGGCAGA
YFV-17D(+)1	Forward	AGTAAATCCTGTGTGCTAATTGAGGTG
ZIKV(+)973	Forward	ATACTTGGTCATGATACTGCT
ZIKV(+)1518	Forward	GGGGTTTTGGAAGCCTAGGA
ZIKV (-)1598	Reverse	GTGAACCAACCAGTGCTTGT
ZIKV(+)1988	Forward	ACGCAGGGACAGATGGAC
YF17D(+)2500	Forward	AGATGGTATCTTCATATTTAGAG
YF17D(+)2800	Forward	GACTTGGGGTAAGAACCTTGTGTTC
YF17D(-)3100	Reverse	GTCACTATGGCTGAGGTGAGA
YFV-17D (-)3725	Reverse	GGGTTACAGCTGGAGAAATACATGC
YF17D(+)3400	Forward	AGTGGGAATATCCCACAAGACA
YF17D(+)3980	Forward	CAGCTTGCCTCAGCAAAGCCTATG
YF17D(-)4600	Reverse	TGGTTGGAGGAGTAGTGCT
YF17D(+)4500	Forward	CTGCCCTCCATCCATTTGC
YF17D(+)5000	Forward	AACAGGAACGGAGAGGTG
YFV-17D (-)5506	Reverse	GCCCCCACCAGGGTTGTTCTTTCT
YFV-17D (+)5249	Forward	TGCTGCGCTTTGATTCCAGGTA
YF17D(+)6000	Forward	ACTATTCTGAGCCTACAAG
YF17D(-)6700	Reverse	ATTGTGCCCATCGCCATAG
YF17D(+)6700	Forward	GAGCCAGGGCAACAAAGGT
YFV-17D(-)7278	Reverse	TGCTGGAGAAAACCAAAGAGGA
YF17D(+)7650	Forward	GAAAAACTTTGGGTGAAG
YF17D(+)8100	Forward	TCACAGAGGGGGAAAGG
YFV-17D(+)8749	Forward	AGTGGTTTTGTGTTTGTCATCCAA
YFV-17D(-)9100	Reverse	TAGGAAGATCATGAAAGTTGTCAACAGG
YF17D(+)9353	Forward	ACATACAAGAACAAAGTGG
YF17D(-)9825	Reverse	ACCACAATCCTCCTGCCATCCTT
YF17D(+)9900	Forward	CAGCTTGCCTCAGCAAAGCCTATG
YF17D(+)10355	Forward	AACACCATCTAACAGGAATAACC
YF17D(-)10566	Reverse	GGCAAAACTCGTGTGGGG
YFV-17D(-)10862	Reverse	AGTGGTTTTGTGTTTGTCATCCAA

Supplementary Table 5: List of all cell lines used in this study

Cell line	Reference/Source
A549	ATCC CCL-185, received from Lieve Naesens (KU Leuven, Belgium)
BHK-21J	Lindenbach & Rice. J Virol 71, 9608–9617 (1997), received from Peter Bredenbeek (LUMC, Leiden, Netherlands)
C6/36	ATCC CRL-1660
HAP-1	Haplogen, Austria (https://www.horizondiscovery.com/cell-lines/all-products/about-x-man-cell-lines/hap1-cell-line-overview)
HEK 293T	ATCC CRL-1573, received from Dirk Daelemans (KU Leuven, Belgium)
Hela	ATCC CCL-2, received from Koen Andries (Johnson & Johnson, Beerse, Belgium)
Huh 7	Nakabayashi et al. Cancer Res, 42, 3858-63 (1982), received from Dominique Schols (KU Leuven, Belgium)
JEG-3	ATCC HTB-36, received from Dominique Schols (KU Leuven, Belgium)
L929	ATCC VR-367, received from Sandra Liekens (KU Leuven, Belgium)
MRC 5	ATCC CCL-171
Raji-DC-SIGN	Geijtenbeek et al. Cell, 100, 587-597 (2000), received from Dominique Schols (KU Leuven, Belgium)
U937	ATCC CRL-1593.2, received from Dominique Schols (KU Leuven, Belgium)
Vero E6	ATCC CRL-1586, received from Peter Bredenbeek (LUMC, Leiden, Netherlands)

Supplementary Table 6: List of all antibodies used in this study

Antibody	Specificity	Validation	Supplier	Catalogue number
**Mouse experiments**				
MAR1-A53	Mouse interferon alpha-beta	Lazear et al., Cell Host Microbe 2016	Leinco Technologies	I-1188
**IIFA**				
D1-4G2-4-15	Pan-flavivirus E	Zmurko et al., Plos Neg Dis. 2016	Millipore	MAB10216
Alexa Fluor-488	Goat anti-mouse H+L	Zmurko et al., Plos Neg Dis. 2016	Life Technologies	A11001
Alexa Fluor 594	Goat anti-mouse H+L	Claus et al., Cereb Cortex, 2018	Life Technologies	A11005
JEV E Ab	JEV E	Arvind and Rangarajan.Biochem Biophys Res Commun. 2016	Life Technologies	MA1-71256
**Flow cytometry**				
eFluor450 anti-mouse CD3e	eBio500A2, eFluor450 anti-mouse CD3e	Fattouh et al., Plos One. 2013	eBioscience	48-0033-82
APC/Cy7 anti-mouse CD8a	53-6.7, APC/Cy7 anti-mouse CD8a	Liu et al., Nature. 2009	BioLegend	100714
PE anti-mouse TNFa	MP6-XT22, PE anti-mouse TNFa	Li et al., Plos One. 2016	BioLegend	506306
APC anti-mouse IFNg	XMG1.2, APC anti-mouse IFNg	Du at al., Nat com. 2016	BioLegend	505810
FITC anti-human/mouse GranzymeB	GB11, FITC anti-human/mouse GranzymeB	Baker et al., Cancer Res. 2014	BioLegend	515403
**Immuno histochemistry**				
Anti-cleaved-caspase-3	Caspase-3	Gladwyn-Ng et al. Nat Neurosci. 2018	Cell Signaling Technology	9661
D1-4G2-4-15	Pan-flavivirus E	Zmurko et al., Plos Neg Dis. 2016	Millipore	MAB10216
Alexa Fluor-488	Goat anti-mouse H+L	Gladwyn-Ng et al. Nat Neurosci. 2018	Life Technologies	A11001
Alexa Fluor-555	Goat anti-mouse H+L	Gladwyn-Ng et al. Nat Neurosci. 2018	Jackson ImmunoResearch Laboratories	A20187
DAPI	Nuclear staining	Gladwyn-Ng et al. Nat Neurosci. 2018	Sigma-Aldrich	28718-90-3
